# High-performance quasi-2D perovskite light-emitting diodes: from materials to devices

**DOI:** 10.1038/s41377-021-00501-0

**Published:** 2021-03-19

**Authors:** Li Zhang, Changjiu Sun, Tingwei He, Yuanzhi Jiang, Junli Wei, Yanmin Huang, Mingjian Yuan

**Affiliations:** Key Laboratory of Advanced Energy Materials Chemistry (Ministry of Education), Renewable Energy Conversion and Storage Center (RECAST), College of Chemistry, Nankai University, 300071 Tianjin, People’s Republic of China

**Keywords:** Inorganic LEDs, Transformation optics

## Abstract

Quasi-two-dimensional (quasi-2D) perovskites have attracted extraordinary attention due to their superior semiconducting properties and have emerged as one of the most promising materials for next-generation light-emitting diodes (LEDs). The outstanding optical properties originate from their structural characteristics. In particular, the inherent quantum-well structure endows them with a large exciton binding energy due to the strong dielectric- and quantum-confinement effects; the corresponding energy transfer among different *n*-value species thus results in high photoluminescence quantum yields (PLQYs), particularly at low excitation intensities. The review herein presents an overview of the inherent properties of quasi-2D perovskite materials, the corresponding energy transfer and spectral tunability methodologies for thin films, as well as their application in high-performance LEDs. We then summarize the challenges and potential research directions towards developing high-performance and stable quasi-2D PeLEDs. The review thus provides a systematic and timely summary for the community to deepen the understanding of quasi-2D perovskite materials and resulting LED devices.

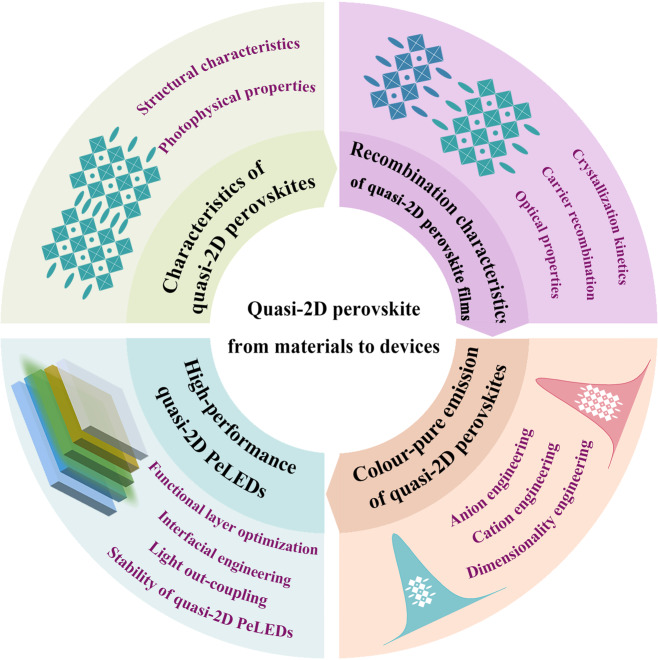

## Introduction

Light-emitting diodes (LEDs) are changing the lighting and display industry and have obtained significant advances compared to traditional lighting sources. Traditional material LEDs, e.g., III–V semiconductor LEDs^1,2^, organic LEDs (OLEDs)^3,4^, and quantum-dot LEDs (QLEDs)^5^, have achieved great success and gradually realized commercialization but still face some challenges. OLEDs have low carrier transport capability and exciton recombination, which hinders the improvement of the brightness. In addition, QLEDs show challenges in terms of the tedious manufacturing process, and the reliance on hydrophobic insulating long ligands also hinders their stability and electrical conductivity. Compared with these traditional materials, metal halide perovskites (MHPs) exhibit superior optoelectronic features that are beneficial for LED applications, such as high photoluminescence quantum yields (PLQYs), narrow full width at half maximum (FWHM), and feasible spectral tunability^6–10^. Perovskite LEDs (PeLEDs) have achieved impressive progress in the past few years since the first room-temperature PeLED was reported in 2014^11^. Three types of perovskite materials with different dimensions (i.e., 3D perovskites, quasi-2D perovskites, and perovskite nanocrystals) are commonly included in the emitter layer of PeLEDs^12–16^. 3D PeLEDs have achieved EQEs of more than 20% in both the near-infrared and green regimes^17,18^. Simultaneously, PeLEDs based on perovskite nanocrystals have also shown prosperous development since they were first reported by Song et al. in 2015^19–21^, achieving a record EQE of 23.4%^22^. Accordingly, the rapid progress achieved in high-performance PeLEDs indicates their promising applications, particularly in ultrahigh-definition displays, solid-state lighting, and photo-communication areas^23,24^.

Quasi-2D perovskites represent an important category of perovskites that possess self-assembled multi-quantum-well structures and have gained great success in light emission applications owing to their outstanding optoelectrical properties^25,26^. Calabrese et al.^27^ demonstrated that MAPbI_3_ (*n* = ∞) perovskite and (RNH_3_)_2_PbI_4_ (*n* = 1) perovskite represent two typical materials in the series of (RNH_3_)_2_MA_*n*−1_Pb_*n*_I_3*n*+1_ (*n* = 1 to ∞). Thereafter, they reported the first quasi-2D perovskite, PEA_2_MAPb_2_I_7_, and the obtained crystallography data unambiguously confirmed the “bilayer” structure. Another pioneering work carried out by Mitzi et al.^28^ highlighted the structural “layered” characteristic of Sn-based perovskites (C_4_H_9_NH_3_)_2_(CH_3_NH_3_)_*n*__−__1_Sn_*n*_I_3*n*+1_ (*n* = 1–5) through crystallographic characterization. Recently, substantial efforts have been made to obtain high-performance quasi-2D PeLEDs, which have facilitated unprecedented rapid development. In the past five years, we have witnessed the rapid development of quasi-2D perovskite optoelectronics, especially their tremendous success in LED applications. The recorded EQE of LEDs has soared to 21% and approached the efficiency limit^22,29^ since the first example was reported in 2016^30^.

In particular, quasi-2D perovskites exhibit unique optical properties arising from their structural characteristics, which are different from those of conventional 3D^11,31–33^ and two-dimensional (2D) perovskites^34,35^. First, quasi-2D perovskites possess natural quantum-well structures, which can induce both dielectric- and quantum-confinement effects^36–40^. Such strong confinements thus afford a large exciton binding energy (*E*_b_). In addition, quasi-2D films feature a mixed-phase rather than a single phase because the formation energies for different quasi-2D phases are quite similar^41^. During photoexcitation, the photocarriers transfer from higher bandgap species to lower bandgap species rapidly and efficiently, leading to accumulated carriers in the recombination centers. The increased carrier density then effectively passivates the defect states, thereby significantly improving the radiative recombination efficiency and the resulting PLQYs^30,42^. In addition, quasi-2D perovskites exhibit tunability of their spectra, which can be modulated through composition and dimensionality engineering respectively. These characteristics enable continuous photoluminescence (PL) wavelength tuning from violet to near-infrared (NIR) spectral regions^29,43–45^.

However, the performance and stability of quasi-2D PeLEDs still cannot meet the requirements for commercialization at the moment. More efforts need to be devoted to exploring the optical and electrical properties of these materials. In addition, investigation of the correlation between the device performance and the underlying photophysics of the materials appears to be particularly important. Following this trend, we discuss the inherent optical properties and corresponding photophysics of quasi-2D perovskites at the beginning of the review. We then summarize the progress in spectral tunability of quasi-2D perovskites, mainly to realize high-performance pure-red and pure-blue emission. Next, we discuss the newly emerged device engineering approaches to produce high-performance quasi-2D PeLEDs. Finally, we summarize the key challenges in the field and propose several promising research opportunities to facilitate the development of highly stable and high-performance quasi-2D materials and devices. The review article thus paves the way for future quasi-2D PeLED manufacture.

## Characteristics of quasi-2D perovskites

### Structural characteristics

**Fig. 1:**
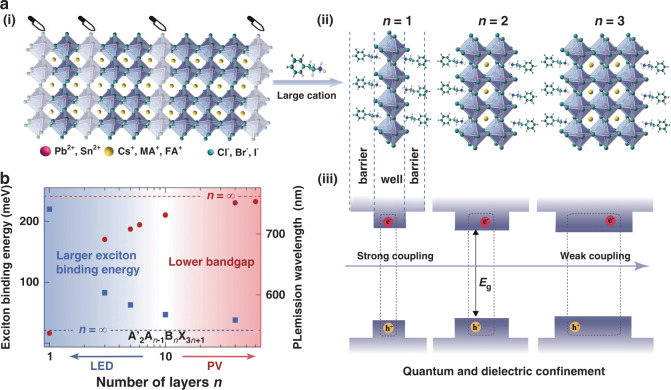
Structure and photophysical properties of a quasi-2D perovskite. **a** (i) Schematic representation of a quasi-2D perovskite, which can be obtained by slicing the 3D perovskite along the <100> crystallographic direction. (ii) Schematic crystal structures of quasi-2D perovskites with different *n*-values. (iii) Electronic properties of quasi-2D perovskites, which are determined by the degree of quantum- and dielectric-confinement effects. **b** *E*_b_ and PL emission wavelength of quasi-2D perovskites as a function of *n*-value. Panel **b** is reprinted from ref. ^51^ with permission from Wiley

Employing bulky organic cations to substantially replace the traditional small cations breaks the original continuous 3D structure and generates a stable quasi-2D geometry. The geometry can be understood as slicing the 3D structure in planes along the <100> crystallographic directions^46–48^. As shown in Fig. 1a, large organic amines are introduced during crystal growth, which cannot enter the gap between [BX_6_]^4^^−^ octahedrons, thus inhibiting the growth of [BX_6_]^4^^−^ along out-of-plane directions^49,50^. The sheets of quasi-2D perovskite unit cells are periodic along the basal plane and are constrained in the perpendicular direction. Generally, quasi-2D perovskites possess the chemical formula A′_2_A_*n*__−__1_B_*n*_X_3*n*+1_ (1 ≤ *n* ≤ ∞), where A′ refers to a large organic cation, including monoammonium cations (R-NH_3_^+^) and diammonium cations (^+^H_3_N-R-NH_3_^+^) (R represents an alkyl chain or aromatic ligand); A stands for a small monovalent cation, e.g., methylammonium (MA^+^ = CH_3_NH_3_^+^), formamidine (FA^+^ = CH(NH_2_)_2_^+^), or cesium (Cs^+^); B is a divalent metal cation such as lead (Pb^2+^) or tin (Sn^2+^); X represents a halide, e.g., chloride (Cl^−^), bromide (Br^−^) or iodide (I^−^); and *n* refers to the number of [BX_6_]^4^^−^ octahedral units. In brief, A′ acts as an insulating layer to isolate the inorganic layers (the metal halide [BX_6_]^4^^−^ octahedral units) linked together by corner-sharing halide anions, and A cations occupy voids within the framework^30,42^.

Quasi-2D perovskites consist of a series of alternately aligned inorganic and organic layers. Inorganic [BX_6_]^4^^−^ octahedral sheets are sandwiched by two layers of large organic spacers with relatively low dielectric constants. Specifically, the inorganic [BX_6_]^4^^−^ slabs act as quantum “wells”, while the organic capping layers function as “barriers”. Thus, the “quantum-well” (QW) structures of a quasi-2D perovskite are formed naturally with an atomically sharp interface between “barriers” and “wells” (Fig. 1a). Due to the quantum- and dielectric-confinement effects arising from the QW structure, the *E*_b_ of a quasi-2D perovskite becomes larger than that of its 3D analog^25,42^. The carrier wave function is compressed in one direction due to the QW width limitation. Accordingly, the carrier movement is limited, which increases the resulting *E*_b_ and effective bandgap of quasi-2D perovskites. In particular, both electrons and holes are confined within the inorganic well; stronger binding energy facilitates the formation of stable excitons at room temperature, thereby increasing the radiative recombination efficiency. Furthermore, the confinement intensity is dependent on the thickness of the QWs, which provides additional flexibility to tune the corresponding bandgap and carrier recombination dynamics^51^ (Fig. 1b). The selection of barriers with different dielectric constants affects the *E*_b_ value, referred to as the “dielectric confinement” effect. Ishihara et al.^52^ noted that the large *E*_b_ (370 meV) was too large to be explained only by the quantum confinement effect. Therefore, the dielectric confinement effect was raised^53^. Kanatzidis et al. simulated a high-frequency dielectric constant (*ε*_∞_) profile for different *n*-values of the BA_2_MA_*n*__−__1_Pb_*n*_I_3*n*+1_ (BA^+^ = CH_3_(CH_2_)_3_NH_3_^+^, MA^+^ = CH_3_NH_3_^+^) family^54^. They demonstrated an increasing *ε*_∞_ for inorganic slabs with increasing *n*-value. The dielectric confinement dominates at *n* = 1, weakens at *n* = 5, and completely disappears in the 3D perovskite (*n* = ∞). Therefore, the dielectric confinement in quasi-2D perovskites also accounts for the corresponding high *E*_b_, and the dielectric confinement decreases as the *n*-value increases (Fig. 1b).

The robustness of the excitonic states at room temperature is the most prominent optical feature of quasi-2D perovskites, which originates from their large *E*_b_. Fortunately, *E*_b_ can be regulated through composition and structure engineering. Basically, incorporating organic cations with different dielectric constants into the quasi-2D structure can significantly tune the dielectric confinement effect^55,56^. In addition, *E*_b_ can also be modulated due to confinement effects by varying the thickness of the QWs^40^. The large *E*_b_ and thus prominent excitonic luminescence are unique features of quasi-2D perovskites with application in LEDs.

### Photophysical properties

Ishihara et al. successively grew quasi-2D single crystals with *n* = 1, 2, 3, and 4^27,28,52^. Afterward, Kanatzidis et al. synthesized and structurally characterized the *n* = 5 (CH_3_(CH_2_)_3_NH_3_)_2_(CH_3_NH_3_)_4_Pb_5_I_16_ perovskite^54^. To date, the maximum *n*-value quasi-2D perovskite reported is the *n* = 7 (CH_3_(CH_2_)_2_NH_3_)_2_(CH_3_NH_3_)_6_Pb_7_I_22_ perovskite^57^. Significantly, the high-quality quasi-2D single-crystal confirms that the structure is thermodynamically stable, which lays the foundation for further optoelectronic applications. The carrier recombination dynamics of quasi-2D perovskite single crystals with various *n*-values were systematically studied to deeply understand the photophysical properties of quasi-2D perovskites.

The carrier recombination dynamics of quasi-2D perovskites can typically be described by the following Eq. (1)^58,59^:

1 $$\frac{{{\mathrm{d}}N(t)}}{{{\mathrm{d}}t}} = - k_1N - k_2N^2 - k_3N^3$$

**Fig. 2:**
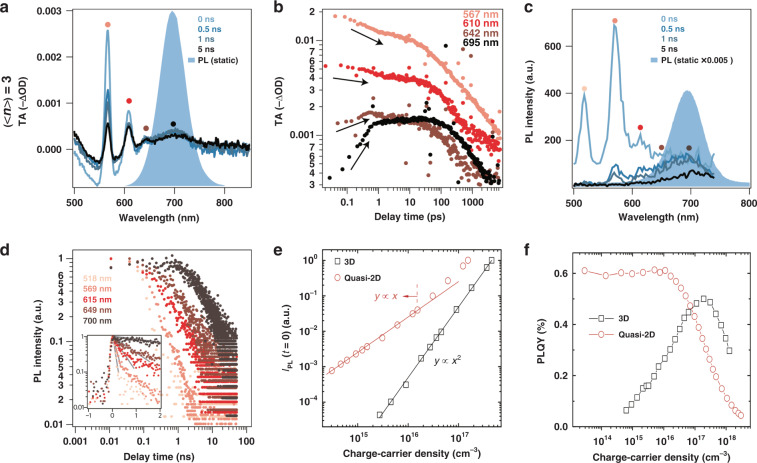
Charge-carrier recombination kinetics in quasi-2D perovskite films. **a** TA spectra at different timescales, **b** TA spectra at different wavelengths as a function of delay time, **c** PL spectra at distinct timescales, and **d** PL decay curve probed at selected wavelengths for <*n*> = 3 perovskites. Comparison of **e** initial time PL intensities and **f** PLQYs as a function of the photoinjected carrier density between 3D and quasi-2D perovskite films. Panels **a**–**d** are reprinted from ref. ^30^ with permission from Springer Nature. Panels **e** and **f** are reprinted from ref. ^62^ with permission from Springer Nature

Here, *N* represents the carrier density at delay time *t*; *k*_1_ refers to the monomolecular recombination constant; *k*_2_ is the bimolecular recombination constant, and *k*_3_ is the three-body Auger (nonradiative) recombination constant. Chen et al.^60^ studied the charge-carrier recombination in quasi-2D perovskite single crystals using transient reflection (TR) spectroscopy. TR kinetics at different excitation fluences were then globally fitted to obtain *k*_1_, *k*_2_, and *k*_3_ for different *n*-value PEA_2_MA_*n*__−__1_Pb_*n*_I_3*n*+1_ crystals. They found that the existence of excitons and free carriers varied in quasi-2D perovskite single crystals with different *n*-values. The largest *k*_1_ was found in the *n* = 1 sample, which can be attributed to its large *E*_b_, indicating that excitons were dominant in this species, while for the *n* = 4 sample, free carriers dominated; for the *n* = 2 and 3 samples, free carriers and excitons coexisted. Additionally, Delport et al.^61^ investigated the recombination dynamics in (C_6_H_5_C_2_H_4_NH_3_)_2_(CH_3_NH_3_)_*n*__−__1_Pb_*n*_I_3*n*+1_ (*n* = 1, 2, 3, and 4) single crystals. They first studied the scaling law of PL_0_ (the PL intensity at *t* = 0 ns, at the instant of pulse excitation) with excitation fluence, which is a classical method used to analyze the recombination behavior. For the *n* = 1 2D single crystal, PL_0_ was linear with the excitation density, showing the predominant exciton recombination characteristic. However, for *n* > 1 single crystals, the nonlinear relationship between PL_0_ and the pump density proved the coexistence of free carrier and exciton recombination. The associated optical and electrical properties seemed to further diverge from those of the pure excitonic compound as the *n*-value increased. To conclude, in low *n*-value quasi-2D perovskites, *E*_b_ is large, which guarantees efficient exciton recombination. In high *n*-value species, the excitons tend to dissociate into free carriers as *E*_b_ decreases (Fig. 2b). The above carrier recombination dynamics in quasi-2D perovskite single crystals have established the potential use of quasi-2D perovskites as optoelectronic materials, such as in solar cells and LEDs.

## Recombination characteristics of quasi-2D perovskite films

### Crystallization kinetics

In light of the efficient excitonic radiative recombination, efforts have been devoted to fabricating high-quality quasi-2D perovskite films, aiming for LED applications^60,62^. The films possess mixed phases with different *n-*values rather than a single phase, which endows them with distinct optical characteristics compared to their single-crystal state^63^. Fortunately, this feature enables efficient energy transfer among different species, which is beneficial for radiative recombination. To better understand and manipulate the formation of the mixed-phase state, the underlying crystallization kinetics in quasi-2D perovskite films should be determined. Quintero-Bermudez et al.^64^ systematically studied the formation process in quasi-2D perovskite films. They found that in the crystallization process, quasi-2D perovskites underwent an intermediate phase state, where tightly combined inorganic slabs, solvents, and organic cations were found. With the evaporation of the solvent, the inorganic layers are newly released from the intermediate phase and then combine with the surrounding organic cations to form a quasi-2D scaffold. The intermediate phase mediated the formation of the quasi-2D structure by providing the scaffold for subsequent nucleation and growth. Consequently, the phase distribution was strongly influenced by whether the organic cations were uniformly distributed on the film.

In particular, selecting different solvents can regulate the distribution of QWs since different cations exhibit various solubilities in different solvents. The kinetics and mechanism of quasi-2D perovskite crystallization can be adjusted by the selection and proportion of the solvent mixture. Compared with dimethylformamide (DMF), dimethyl sulfoxide (DMSO) can form strong Lewis base adducts with lead halide^65^ and strong hydrogen bonds with ammonium salts due to its high polarity (7.2)^66^. Thus, the presence of DMSO further increased the nucleation barrier of the perovskites. *n* < 4 species were remarkably favored when intermediate phases transformed into quasi-2D perovskites owing to the intrinsic lower nucleation barrier compared with *n* > 4 species^66^. Therefore, the mixed solvent or neat DMSO increased the disparity of nucleation barriers among various phases and thus led to a broader phase distribution compared to the neat DMF case^67^.

### Carrier recombination characteristics

Energy transfer in quasi-2D perovskite films originates from the graded band structure because of mixed *n*-value species. This structure acts as a carrier concentrator, increasing the carrier density of the recombination center. The resulting high carrier density thus partially photopassivates the shallower trap states, thereby significantly avoiding trap-mediated nonradiative recombination^25,30,42^. The energy transfer facilitates radiative recombination, resulting in high PLQYs for quasi-2D perovskite films even at low pumping densities. Thus, profoundly understanding the energy transfer kinetics and effectively modulating them are demanded to construct efficient quasi-2D optoelectronics.

Yuan et al.^30^ carried out ultrafast spectroscopy to investigate the carrier recombination kinetics for (PEA)_2_MA_*n*__−__1_Pb_*n*_I_3*n*+1_ quasi-2D perovskite films. Intriguingly, the TA spectra exhibited four distinctive bleaching peaks in <*n*> = 3 (<*n*> represents the average “QW” thickness) films ascribed to *n* = 2, 3, 4, and 5 species. Figure 2a shows the relative intensity evolution of these bleaching peaks. The data demonstrated that carriers transfer from small *n*-value species to large *n*-value species. The build-up time for GSB of lower bandgap species was in good agreement with the fast decay time of higher bandgap species, which was less than 1 ps and indicated that the energy transfer was ultrafast (Fig. 2b). Time-resolved photoluminescence (TRPL) measurements revealed the same trend (Fig. 2c). Specifically, the lower bandgap species exhibited a biexponential decay, and the corresponding fast component was attributed to carrier funneling from large bandgap species (Fig. 2d).

Xing et al.^62^ investigated the power-dependent initial PL intensity (*I*_PL_ [*t* = 0]) for (NMA)_2_FA_*n*__−__1_Pb_*n*_I_3*n*+1_ quasi-2D perovskite films (Fig. 2e). Notably, *I*_PL_ [*t* = 0] was linear with excitation density below 1.5 × 10^16^ cm^−^^3^, while a clear transition from linear to superlinear was observed when the excitation density increased continuously. They demonstrated that monomolecular radiative exciton recombination was dominant under a low carrier density and gradually changed to free electron-hole bimolecular recombination as the carrier density increased further. Consequently, the PLQY of a quasi-2D perovskite can be given by the following equation^62^:

2 $${\mathrm{PLQY}}\left( N \right) = \frac{{{\sum} {k_{\mathrm{r}}} }}{{{\sum} {k_{\mathrm{r}} + {\sum} {k_{{\mathrm{nr}}}} } }} = \frac{{k_{1,{\mathrm{exciton}}} + k_2N}}{{k_{1,{\mathrm{exciton}}} + k_{1,{\mathrm{trap}}} + k_2N + k_3N^2}}$$

Here, the monomolecular recombination constant *k*_1_ contains both *k*_1,exciton_ and *k*_1,trap_, where *k*_1,exciton_ is the radiative exciton recombination constant and *k*_1,trap_ is the nonradiative trap-assisted recombination constant. The PLQY only depends on two physical processes, namely, radiative recombination (*k*_r_) and nonradiative recombination (*k*_nr_), and is the result of competition between these two channels. Specifically, for quasi-2D perovskites, radiative recombination includes exciton recombination (*k*_1,exciton_) and free carrier recombination (*k*_2_); nonradiative recombination includes trap-assisted recombination (*k*_1,trap_) and Auger recombination (*k*_3_). In addition, these recombination rate constants (*k*_1_, *k*_2_, and *k*_3_) strongly depend on the carrier concentration (*N*). Therefore, the PLQY of quasi-2D perovskite films is dependent on *N*. At low *N*, PLQY only depends on the competition between *k*_1,exciton_ and *k*_1,trap_. Fortunately, the high PLQY and near invariant dependence for quasi-2D perovskite films at carrier densities below 10^16^ cm^3^ validated that radiative exciton recombination overwhelmed trap-mediated nonradiative recombination^62^ (Fig. 2f). Additionally, many defect passivation strategies are used to reduce *k*_1,trap_. At high *N*, *k*_3_ increases sharply and gradually dominates, resulting in a decrease in PLQY^68^. Therefore, optimizing the optical properties of quasi-2D perovskite films mainly involves increasing *k*_1,exciton_ and *k*_2_, and simultaneously suppressing *k*_1,trap_ and *k*_3_.

### Optical property modulation

From the perspective of the crystallization kinetics and the carrier recombination characteristics of quasi-2D perovskite films, we conclude that efficient energy transfer, effective exciton recombination, and low defect density are the most striking features for quasi-2D perovskite films, which contribute to the excellent optical properties^69^. Here, we summarize the widespread strategies towards highly emissive quasi-2D perovskite films from the three aspects above.

**Fig. 3:**
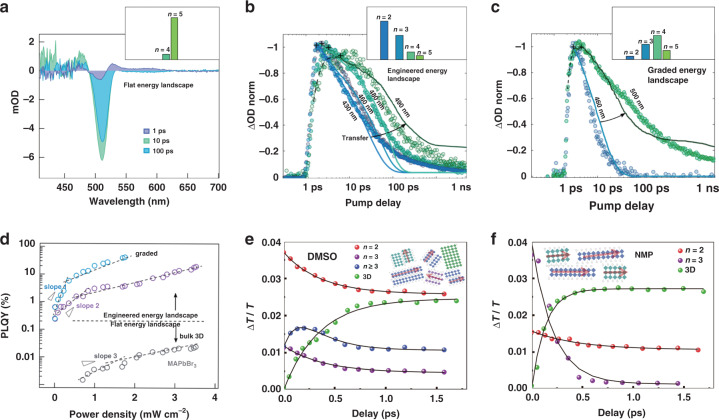
Phase distribution and crystal orientation for effective energy funneling. **a** TA spectra at different timescales for a quasi-2D perovskite film with a flat energy landscape. Time-dependent TA spectral traces for **b** an <*n*> = 3 perovskite film with an engineered energy landscape and **c** an <*n*> = 5 film with a graded energy landscape. **d** Pump power density-dependent PLQYs for different perovskite systems. Time-dependent TA spectral traces of different quasi-2D perovskite films obtained from **e** DMSO and **f** NMP solvents. Panels **a**–**d** are reprinted from ref. ^75^ with permission from the American Chemical Society. Panels **e** and **f** are reprinted from ref. ^77^ with permission from Wiley

Efficient energy transfer requires photogenerated carriers to transfer quickly to lower bandgap species to escape from the trapping process. Therefore, energy transfer pathway optimization is highly needed to realize high-efficiency energy transfer^70^. For quasi-2D perovskite films, the *n*-value distribution affects their energy transfer and corresponding radiative exciton recombination efficiency. Fortunately, the *n*-value distribution can be modulated through fabrication process engineering^71–74^. Quan et al. tailored the energy landscapes in PEA_2_(MA)_*n*__−__1_Pb_*n*_Br_3*n*+1_ perovskite using antisolvent engineering. As shown, the flat energy landscape in the <*n*> = 3 film led to subtle energy transfer, whereas the graded energy landscape in the <*n*> = 5 films, which consisted of different *n*-value species, facilitated the resulting energy transfer (Fig. 3a, b). They then optimized the concentration of *n* = 5 species in the <*n*> = 5 film to make a graded energy landscape, which can make energy transfer more efficient^75^ (Fig. 3c). As a result, a high PLQY (60%) was achieved in the <*n*> = 5 films with the graded energy landscape (Fig. 3d). In addition, recent studies have noted that the grain orientation can also affect energy transfer efficiency. Lei et al.^76^ demonstrated that a highly oriented quasi-2D perovskite film exhibited a faster Förster resonance energy transfer (FRET) than a randomly oriented film due to the decreased donor-acceptor distance and aligned dipole orientation^77^. Using *N*-methyl-2-pyrrolidone (NMP) as the solvent, PEA_2_(FA)_*n*__−__1_Pb_*n*_Br_3*n*+1_ films with grains highly parallel to the substrate were obtained, while randomly oriented films were achieved by using DMSO as the solvent. Consequently, more efficient energy transfer was realized in the highly oriented quasi-2D perovskite films according to various optical characterizations (Fig. 3e, f). In addition to the *n*-value distribution and the grain orientations, the coupled quasi-2D perovskite phases can also affect the energy transfer. Ren et al. introduced a bifunctional ligand (4-(2-aminoethyl) benzoic acid, ABA) into the mixed-ligand perovskite PEA/PA(CsPbBr_3_)_*n*__−__1_PbBr_4_ to promote coupled quasi-2D perovskite phases^40^. The strengthened interaction between the coupled perovskite phases would benefit efficient energy transfer in films, resulting in prolonged operational stability in PeLEDs.

**Fig. 4:**
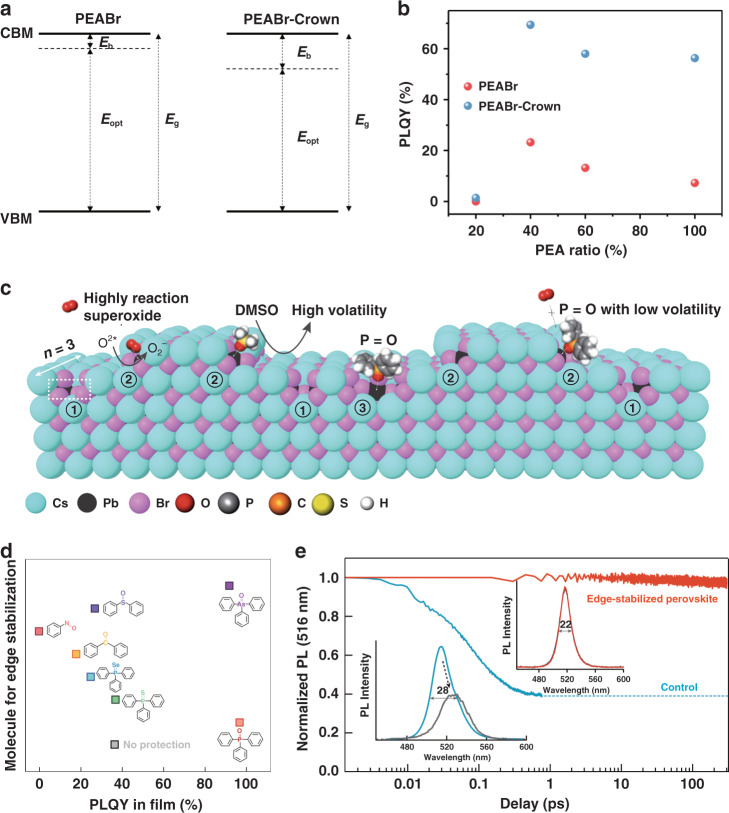
Additive engineering for high-efficiency and stable quasi-2D perovskite films. **a** Energy schematic diagram of 40% PEABr with and without a “crown” additive. **b** PLQY versus PEABr ratio for the quasi-2D perovskite films with and without the “crown” additive. **c** Schematic illustrating imperfect edges of PEA_2_Cs_2_Pb_3_Br_10_ perovskite films and the reaction pathway to produce superoxide under photoexcitation. **d** Corresponding PLQYs of quasi-2D perovskite films treated with different molecules. **e** PL stability for the control (blue) and edge-stabilized (red) quasi-2D perovskites before and after (in gray) measurement. Panels **a** and **b** are reprinted from ref. ^78^ with permission from Springer Nature. Panels **c**–**e** are reprinted from ref. ^79^ with permission from Springer Nature

Enhancing radiative exciton recombination by increasing *E*_b_ can also improve the optical properties of quasi-2D perovskite films. Ban et al. demonstrated that severe phase separation between the perovskite and the organic phase would weaken dielectric confinement, introducing nonradiative recombination channels. Therefore, they used a crown molecule as an additive to suppress the π–π stacking between PEA cations, thereby inhibiting phase separation^78^. The suppression of phase separation led to a more pronounced dielectric confinement effect and an increased *E*_b_ (69.5 meV) (Fig. 4a). The *k*_1_ fitted by TA dynamics for crown-treated quasi-2D perovskite films showed a value of 9 × 10^−6^ s^−1^, which was 1.5 times that of the controls without the crown. As a result, the crown-treated quasi-2D perovskite films exhibited an enhanced PLQY of 70 ± 8% compared with the pristine films (23 ± 5%) (Fig. 4b). However, with increasing *E*_b_, the enhancement of exciton recombination is accompanied by increases in *k*_2_ and *k*_3_. Therefore, the acquisition of a high radiative recombination efficiency should consider the trade-off between the high radiative recombination constant (*k*_1,exciton_ and *k*_2_) and the low nonradiative recombination constant (*k*_1,trap_ and *k*_3_).

According to Eq. (2), minimizing *k*_1,trap_ is equally important for obtaining high PLQYs of quasi-2D perovskite films. Owing to the ultrafast energy transfer process, quasi-2D perovskite films have less first-order nonradiative recombination loss than their bulk 3D analogs^67^. However, due to many uncontrollable factors during nucleation and growth, trap states still exist in the films. Generally, the generation of trap states is related to the volatilization of the solvent during the crystallization process. Specifically, Lewis base polar aprotic solvents, such as DMSO, DMF, or NMP, which readily form intermediate phases with metal halides, are widely used in the dissolution of perovskite precursors and the control of the crystallization rate^79^. However, the Lewis base metal complexes decompose under the annealing process with the evaporation of the solvent. Then, metal dangling bonds and halogen vacancies are inevitably brought to the grain surface. The undesirable edge states are sensitive to moisture and oxygen, where additional low-level orbitals can be provided once an oxygen atom is adsorbed. These edge states thus further serve as exciton capture sites. Small molecule additives with lone-pair electrons can provide strong bonding with Pb dangling bonds and reduce the density of halogen vacancies^80^. Yang et al.^81^ used Lewis base trioctylphosphine oxidation (TOPO) to passivate the surface of a (PEA)_2_FA_2_Pb_3_Br_10_ film. After the surface treatment, the PLQY of the film increased from 57.3% to 73.8%. They addressed that the passivation effect originated from the bond between the P=O group in TOPO and the incomplete [PbBr_6_]^4^^−^ octahedra. Quan et al.^79^ found that the rapid photodegradation of quasi-2D perovskite films arose from edge-initiated oxidation. Photodegradation occurred as photogenerated carriers diffused to the edge states and produced superoxide (Fig. 4c). Therefore, they adopted an edge-stabilization strategy in which triphenylphosphine oxides passivated the halogen vacancy traps. With this strategy, the passivated quasi-2D films obtained an edge-stable state and showed a near-unity PLQY up to 97% (Fig. 4d). Notably, small molecules including P=O or As=O groups showed strong binding energies with unsaturated Pb in quasi-2D perovskite films. More importantly, the perovskite films maintained excellent stability. After continuous illumination for more than 300 h in ambient air, no significant drop in PL intensity or emission peak shift was observed (Fig. 4e). In addition to small molecules, Lewis base polymers such as polyethylene oxide (PEO) and polyethylene glycol (PEG) also have an effective passivation effect on halogen vacancy traps^82–85^. Such polymers contain a large number of oxygen atoms with lone-pair electrons. They can coordinate with Pb^2+^ to form passivation layers on the perovskite grain surface, which significantly reduces trap-assisted nonradiative recombination. Additionally, these polymers could reduce the grain size, improve the film quality, inhibit ion migration, and enhance the stability of quasi-2D perovskite films.

Precursor composition engineering is another effective method to reduce the trap state density of thin films. Since the crystallization of quasi-2D perovskite films is a self-assembly process, the precursor composition strongly influences the surface state, grain boundaries, and phase distribution of the ultimate films. Cheng et al.^86^ fabricated quasi-2D perovskite films via two different precursor compositions: stoichiometric (ST) and extensive organic cation-doped (LOD). Compared with the ST precursor, the nonstoichiometric LOD precursor possessed a high organic cation/Pb^2+^ ratio, thus providing more PEA^+^ to passivate the grain boundaries and avoiding the formation of the unfavorable low-*n* phase. As a result, the LOD perovskite films showed remarkable optical properties, with the highest PLQY up to 95.3%.

## Color-pure emission of quasi-2D perovskites

**Fig. 5:**
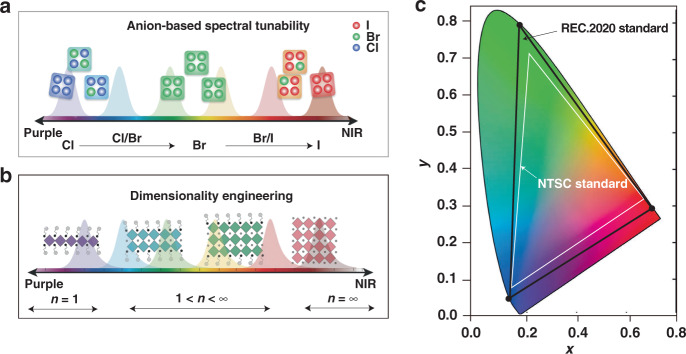
Spectral tunability of quasi-2D perovskites. Schematic representation of spectral tunability in quasi-2D perovskites realized by **a** anion and **b** dimensionality engineering. **c** CIE chromaticity coordinate diagram for display color gamut showing the Rec. 2020 and NTSC standards. Panels **a**, **b** are reprinted from ref. ^51^ with permission from Wiley

Quasi-2D perovskites can achieve emission from violet to NIR spectral regions by chemical composition adjustment and dimensionality engineering (Fig. 5a, b). To date, EQEs exceeding 20% have been successfully achieved in green and NIR quasi-2D PeLEDs, while the realization of high-performance pure red and blue quasi-2D PeLEDs still encounters many obstacles. The most advanced Recommendation BT 2020 (Rec. 2020) standard demands that the monochromatic RGB primaries should approach (0.708, 0.292), (0.170, 0.797) and (0.131, 0.046) for red, green and blue in Commission Internationale de L’Eclairage (CIE) coordinates^87^ (Fig. 5c). Simultaneously, a narrow full width at half maximum (FWHM) (<25 nm) is required. However, the pure red and blue quasi-2D PeLEDs still show lower efficiency, color purity, and stability than the desired values^84,88–91^. Here, we summarize three promising strategies to achieve high-performance pure red and blue quasi-2D PeLEDs.

### Anion engineering

**Fig. 6:**
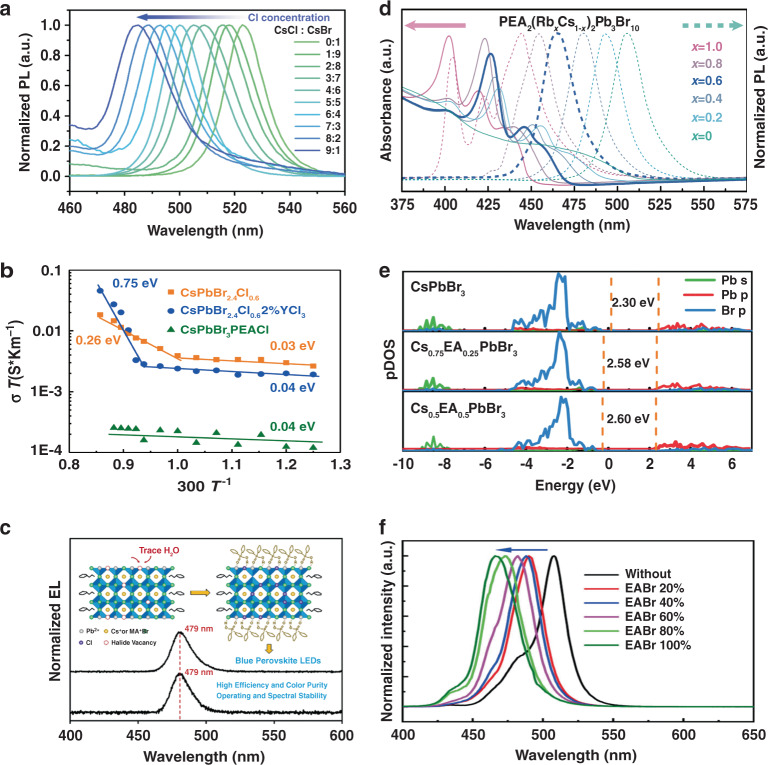
Tuning the spectra via anion and cation engineering. **a** PL spectra of 3D perovskite films with various contents of Cl ions. **b** Temperature-dependent conductivity measurement for different perovskite films to obtain the activation energy of ion migration. **c** EL spectra of control and DPPOCl-treated quasi-2D PeLEDs. Inset: Schematic representation of Cl ion insertion and immobilization in DPPOCl-treated perovskites. **d** UV-vis absorption and PL spectra of PEA_2_(Rb_*x*_Cs_1−*x*_)_2_Pb_3_Br_10_ perovskites with various contents of Rb ions (0 ≤ *x* ≤ 1). **e** Calculated electronic density of states (DOS) of control and EABr-treated perovskites. **f** PL spectra for different EABr contents. Panel **a** is reprinted from ref. ^97^ with permission from Springer Nature. Panel **b** is reprinted from ref. ^102^ with permission from Springer Nature. Panel **c** is reprinted from ref. ^104^ with permission from the American Chemical Society. Panel **d** is reprinted from ref. ^110^ with permission from Springer Nature. Panels **e**, **f** are reprinted from ref. ^45^ with permission from Springer Nature

Anion engineering is a straightforward strategy to achieve pure red and blue emission^92–96^. For instance, Li et al. reported blue emission perovskite films by partial substitution of Br^−^ with Cl^−^ in PEA_2_(CsPbBr_3_)_*n*__−__1_PbBr_4_ perovskite (Fig. 6a). However, the resulting quasi-2D PeLEDs presented spectral instability when the applied voltage exceeded 6 V. This spectral instability resulted from phase separation due to the migration of Cl^−^ and Br^−^ under the electric field^97^. A similar phenomenon was also observed in red quasi-2D PeLEDs with mixed bromide-iodide perovskite films^98^.

Strategies have been developed to suppress the undesired ion migration in mixed-halide quasi-2D perovskite systems^99,100^. Previous experience with perovskite photovoltaics suggested that the free energy linked to the composition or electronic structure can drive ion migration^101^. Li et al. reported a spectrally stable blue quasi-2D film by adding 2% yttrium(III) chloride (YCl_3_) to the precursor. The improved spectral stability can be attributed to inhibited ion migration. The incorporation of yttrium increased the ion migration activation energy of the quasi-2D perovskite from 0.26 to 0.75 eV, making it more thermodynamically stable^102^ (Fig. 6b). In addition, theoretical calculations and experimental results suggested that phase separation could be mediated by ionic defects, especially by halide vacancies^103^. Thus, defect passivation can be used to mitigate or eliminate ion migration. Recently, Sargent et al. reported that the treatment of a PEA_2_Cs_1.6_MA_0.4_Pb_3_Br_10_ film with diphenyl phosphine chloride (DPPOCl) achieved stable blue emission^104^. They noted that DPPOCl would first react with trace water and release Cl^−^ to saturate the halide vacancies. Meanwhile, DPPOCl would form hydrogen bonds to in situ immobilize the inserted Cl^−^ (Fig. 6c). The chloride insertion-immobilization strategy enabled bright, narrowband, and stable blue quasi-2D PeLEDs. In addition, a recent study also proposed that local strains induced by lattice mismatch could facilitate ion migration^105^. An avenue was thus presented to enhance the intrinsic stability of perovskite films by reducing the residual strain in films. The above findings indicate that it is feasible to realize pure red and blue quasi-2D PeLEDs by using anion engineering, but the relevant studies are insufficient, and further research is required. Further research should focus on exploring more effective ion stabilization strategies to achieve more spectrally stable quasi-2D PeLEDs.

### Cation engineering

Spectra can also be tuned through “A-site” or “B-site” substitutions in quasi-2D perovskites. Commonly used “A-site” cations, e.g., Cs^+^, MA^+^, and FA^+^, possess incremental ionic radii (*R*) (*R*_Cs+_ = 1.67 Å, *R*_MA+_ = 2.70 Å, *R*_FA+_ = 2.79 Å). The ion radius change causes the perovskite lattice to deviate from the desired tolerance factor, resulting in structural distortion and bandgap alteration^106–108^. Previous reports demonstrated that introducing Rb^+^ (R_Rb+_ = 1.52 Å) into a perovskite resulted in a significant increase in the bandgap due to the tilt of the inorganic octahedron and the reduction in orbital overlap^109^. The bandgap of Rb_*x*_Cs_1__−__*x*_PbBr_3_ perovskite films increased from 2.31 to 2.60 eV (0 ≤ *x* ≤ 0.8) with increasing Rb^+^. Jiang et al.^110^ partially substituted Cs^+^ with Rb^+^ and fabricated alloy PEA_2_(Rb_*x*_Cs_1__−__*x*_)_2_Pb_3_Br_10_ films. The small-radius Rb^+^ increased the optical bandgap of these films and realized blue emission within the range of ~450–490 nm (Fig. 6d). Moreover, alloy PEA_2_(Rb_*x*_Cs_1__−__*x*_)_2_Pb_3_Br_10_ films exhibited impressive spectral stability compared with the mixed-halide films since the undesired halide migration or Ostwald ripening had been overcome. Recently, Chu et al. used EA^+^ (CH_3_CH_2_NH_3_^+^) to partially replace Cs^+^ and achieved pure-blue emission in PEA_2_(EA_*x*_Cs_1__−__*x*_PbBr_3_)_2_PbBr_4_ perovskite. They claimed that the incorporation of EA^+^ could decrease the Pb-Br orbital coupling and increase the bandgap (Fig. 6e). This strategy modulated the PL peak from the green region (508 nm) to the blue region (466 nm) with increasing EA^+^ (Fig. 6f), and over 70% PLQY in blue emission was obtained^45^. Lanzetta et al.^111^ reported the 2D perovskite materials (PEA)_2_SnI_*x*_Br_4__−__*x*_ with tunable optical properties in the visible spectral region. Limited to the manufacturing technology at that time, they only fabricated PeLEDs with extremely poor performance at 630 nm. Subsequently, Yuan et al.^112^ developed a strategy to improve the film quality and protect Sn^2+^ from oxidation by adding valeric acid (VA). They fabricated color-pure red PEA_2_SnI_4_ LEDs with an EQE of 5% and a lifetime of >15 h.

In conclusion, cation engineering of the “A-site” or “B-site” is another feasible strategy to achieve pure red and blue emission. This strategy dramatically slows down the spectral redshift caused by halogen segregation, thus showing excellent application potential in long-term stable quasi-2D PeLEDs. The “A-site” and “B-site” options are still limited, and researchers should exploit more suitable inorganic or organic cations to provide further breakthroughs.

### Dimensionality engineering

Quasi-2D perovskites with high structural tunability can enable flexible regulation of the quantum-confinement effect. Reducing the average <*n*> value of the film enhances the quantum-confinement effect, broadens the perovskite bandgap, and results in spectral blueshift. Thus, dimensionality engineering offers an efficient approach for spectral manipulation^86,97^. The average <*n*> values of quasi-2D perovskite films are determined by the equilibrium between the large organic cations and the precursor^88^. In theory, increasing the content of large organic cations can monotonically reduce the <*n*> value of quasi-2D perovskite films. However, this does not mean that quasi-2D perovskite films with low <*n*> values are sufficient to effectively realize pure red or blue emission. For instance, increasing the content of large organic cations results in excessive generation of a low *n*-value phase, which leads to inefficient energy transfer and reduced optical properties^113^. Meanwhile, poor charge transport properties arise from large amounts of insulating organic cations. Moreover, the strong electron–phonon coupling and exciton–exciton annihilation at small <*n*> values act as nonradiative recombination pathways and further deteriorate the optical properties^114^.

**Fig. 7:**
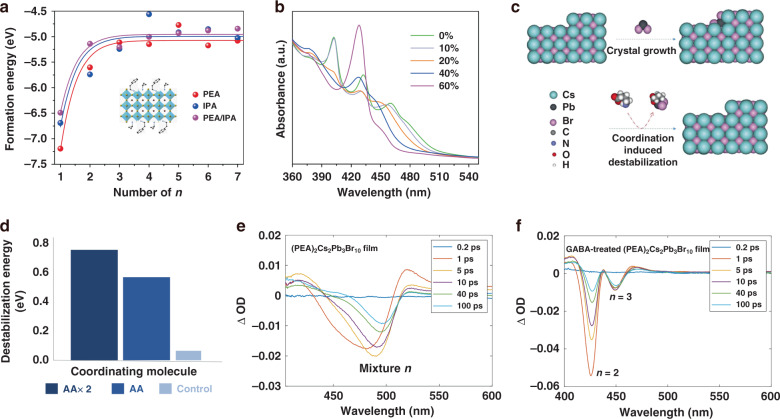
Phase modulation of quasi-2D perovskites for effective blue emission. **a** Calculated formation energy of PEABr- and IPABr-based perovskites and their mixed quasi-2D perovskites with different *n-*values. Inset: Atomic model of the PEA/IPA mixed quasi-2D perovskite with *n* = 3. **b** UV-vis absorption spectra of the perovskite PEA_2_A_1.5_Pb_2.5_Br_8.5_ with different amounts of IPABr additive. **c** Schematic diagram of the chelating effect on PbBr_2_ binding to the surface. **d** DFT-calculated destabilization energy of PbBr_2_ on the quasi-2D perovskite surface when coordinated with GABA and PEA. TA spectra at different timescales for **e** (PEA)_2_Cs_2_Pb_3_Br_10_ and **f** GABA-treated (PEA)_2_Cs_2_Pb_3_Br_10_ perovskite films. Panels **a** and **b** are reprinted from ref. ^121^ with permission from Springer Nature. Panels **c**–**f** are reprinted from ref. ^122^ with permission from Springer Nature

Judicious phase modulations towards a narrow phase distribution are highly desired to realize pure red and blue emission, which would address the severe optical property degradation in small <*n*> value films^44,115–120^. Controlling crystallization by antisolvent techniques or rational large cation spacers and additives can narrow the phase distribution^72,115^. Xing et al. selected the short organic cation isopropylammonium (IPA^+^) to partially replace the longer cation (PEA^+^) in PEA_2_A_1.5_Pb_2.5_Br_8.5_ (A = MA^+^ and Cs^+^) films, which can modulate the crystallization and phase distribution in the quasi-2D perovskite. Theoretical calculations showed that the formation energy of the *n* = 1 phase changed from −7.2 (more stable) to −6.5 eV (less stable) when these two cations were used synergistically (Fig. 7a). Thus, increasing the IPA^+^/Pb^2+^ ratio suppressed the formation of the *n* = 1 phase and inhibited high-*n* phase generation afterward, while the intermediate *n* phases (*n* = 2, 3, 4) grew faster instead (Fig. 7b). Simultaneously, the PL peaks blueshifted from 497 to 467 nm as the IPA^+^/Pb^2+^ ratio increased from 10 to 60%^121^. As a result, the resultant films displayed high PLQYs and stable blue emission by modulating the phase judiciously, thereby fabricating efficient and spectrally stable sky-blue quasi-2D PeLEDs. Since then, the effect of mixed organic cations on the properties of quasi-2D perovskites has been extensively studied.

In addition to mixed-cation strategies, a judicious phase distribution can be achieved by rationally screening additives. Wang et al.^122^ incorporated a chelating agent, γ-aminobutyric acid (GABA), into a PEA_2_Cs_*n*__−__1_Pb_*n*_Br_3*n*+1_ film. Theoretical calculations indicated that the coordination tendency of small chelating molecules towards PbBr_2_ in the vicinity of the perovskite could inhibit the binding of PbBr_2_ to the perovskite surface, suppressing the growth of the large *n* phase. Replacing the unidentate group (PEA^+^) with a small bidentate molecule (GABA) resulted in a 10-fold increase in the destabilization energy (0.51 eV), which increased further when two GABA molecules were utilized for coordination (Fig. 7c, d). The photoexcited carrier dynamics of the GABA-treated quasi-2D perovskite films adequately proved that the resulting phase distribution was concentrated at *n* = 2 and 3 (Fig. 7e, f). The efficient energy transfer from the judicious phase distribution of the films can increase the PLQY and realize true-blue emission (EL at 478 nm)^122^. In conclusion, after a series of artificial designs and interventions regarding the phase distribution, the optical properties of pure red and blue quasi-2D perovskite films have significantly improved, paving the way for high-performance pure red and blue quasi-2D PeLED manufacture. In our opinion, successful fabrication of high-performance quasi-2D PeLEDs with pure red and blue emission that satisfy display purposes might require a combination of strategies leveraging anion engineering, cation engineering of the “A-site” or “B-site,” and dimensionality engineering.

## High-performance quasi-2D PeLEDs

Highly emissive perovskite layers are not sufficient to obtain high-performance quasi-2D PeLEDs due to the difference between photoluminescence and electroluminescence. The working principle and important parameters of PeLEDs need to be specifically considered. PeLEDs can be simplified into a double-heterojunction structure, in which the perovskite emitter layer is sandwiched between the *p*-type hole transport layer (HTL) and the *n*-type electron transport layer (ETL)^123^. Under a forward voltage, holes and electrons are injected from the anode and cathode, respectively, and are confined in the perovskite layer. Then, the holes and electrons release photons through radiative recombination. The key parameters, including the EL peak, FWHM, luminance, turn-on voltage (*V*_on_), EQE, and operational stability, are used to evaluate the performance of PeLEDs^124^ (Table 1). For display devices with a wide color gamut, LEDs usually need to have a specific EL peak and a narrow FWHM to achieve emission purity, while in the case of white-light devices for solid-state lighting, the devices have a wide emission range and FWHM. The luminances of LEDs are usually between 200 and 1000 cd m^−2^ for display applications and exceed 10,000 cd m^−2^ for solid-state lighting^125^. *V*_on_ refers to the voltage when the luminance of the device reaches 1 cd m^−2^. A low *V*_on_ represents an effective injection of carriers. The operational stability of PeLEDs is usually evaluated by *T*_50_, which represents the time for the luminance to drop to half of its initial value when working at a fixed current or voltage.

EQE is defined as the ratio of the number of photons emitted by the device to the number of electrons injected and is the most important indicator for judging the energy conversion efficiency of LEDs. EQE can be expressed as the product of the internal quantum efficiency (IQE) and light extraction efficiency (*η*_oc_)^126^.

3 $${\mathrm{EQE}} = {\mathrm{IQE}} \times \eta _{{\mathrm{oc}}} = \gamma \times \chi \times \eta _{{\mathrm{PL}}} \times \eta _{{\mathrm{oc}}}$$

Here, IQE is defined as the ratio of the number of photons generated to the number of electrons injected into the LED; *η*_oc_ represents the ratio of the number of photons emitted to the outside to the number of photons generated in the active layer; *γ* represents the charge injection balance factor; *χ* refers to the fraction of excitons for radiative decay, and *η*_PL_ is the PLQY^127^. *η*_PL_ has been detailed before, and *η*_oc_ will be elaborated below. Here, we focus on *γ* and *χ*, which relate to the device structure and electrical factors. Device engineering, such as optimization of the charge transport layers of quasi-2D PeLEDs, could promote the charge injection balance factor towards its maximum (*γ* = 1)^128^. In addition, the use of electron- and hole-blocking layers can confine the charge carriers in the emitting layer and thus lead to enhanced charge balance. The use of interfacial engineering to reduce the exciton quenching at each interface of the device could promote the fraction of excitons for radiative decay (*χ*)^129^. Based on Eq. (3), we summarize three aspects to improve the electrical properties in quasi-2D PeLEDs, including function layer modulation, interfacial engineering, and light out-coupling technologies. Finally, the operational stability is another critical parameter of quasi-2D PeLEDs, and we then overview several possible reasons for degradation^130^.

### Functional layer optimization

**Fig. 8:**
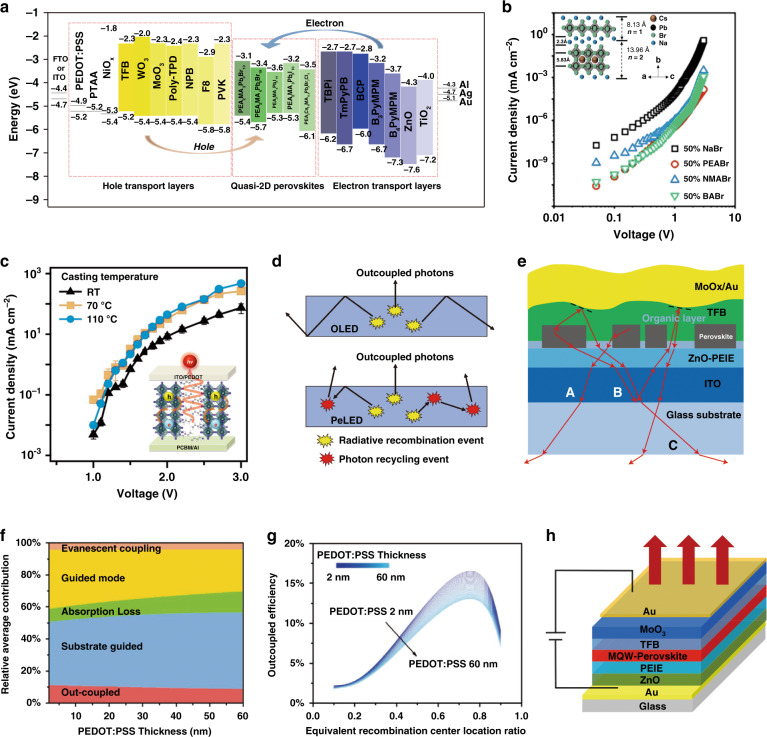
Device engineering and characterization of quasi-2D PeLEDs. **a** Energy levels for different transport layers and emitting layers. **b** *J*–*V* curves for hole-only devices based on quasi-2D perovskite films with different spacer cation salts. Inset: Unit cell of Na_2_Cs_*n*−1_Pb_*n*_Br_3*n*+1_ perovskites with *n* = 1 and *n* = 2. **c** *J–V* curves for (BA)_2_(MA)_2_Pb_4_I_13_ PeLEDs cast at different temperatures. Inset: Scheme illustrating the charge injection and recombination processes in oriented film. **d** Schematic diagram for photon recycling in PeLEDs and OLEDs. **e** Formation of submicrometric structures in PeLEDs to enhance the light out-coupling efficiency. **f** Power dissipation channels of PeLEDs determined by the PEDOT:PSS layer thickness. **g** Light extraction efficiency changes with equivalent recombination center location for various PEDOT:PSS layer thicknesses. **h** Device structure of top-emission PeLEDs. Panel **b** is reprinted from ref. ^43^ with permission from the American Chemical Society. Panel **c** is reprinted from ref. ^139^ with permission from Wiley. Panel **d** is reprinted from ref. ^155^ with permission from Wiley. Panel **e** is reprinted from ref. ^17^ with permission from Springer Nature. Panels **f** and **g** are reprinted from ref. ^159^ with permission from Wiley. Panel **h** is reprinted from ref. ^29^ with permission from Springer Nature

To convert high PLQYs of quasi-2D perovskite films into high EQEs of quasi-2D PeLEDs, the band alignment of the device structure is the most basic consideration. Typically, PeLEDs have a sandwich device structure in which the perovskite emissive layer is located between the electron and hole transport layers. Ideally, the charge balance factor can be maximized to 1 (*γ* = 1) by optimizing the charge transport layer. The energy levels for different transport layer materials (TLMs), including HTLs and ETLs, are shown in Fig. 8a. Appropriate TLMs should have ideal energy levels for efficient carrier transport while blocking opposite carrier transport. In addition, the carrier mobility of different TLMs also affects the carrier injection balance. For ETLs, PO-T2T (2,4,6-tris[3-(diphenylphosphinyl) phenyl]-1,3,5-triazine) can enable overall performance improvements compared to B3PYMPM (4,6-bis(3,5-di(pyridin-3-yl) phenyl)-2-methylpyrimidine) and TPBi (2,2′,2″-(1,3,5-benzinetriyl)-tris(1-phenyl-1-H-benzimidalzole))^131^. The deeper HOMO level (−7.5 eV) and the superior electron mobility (∼10^−3^ cm^2^ V^−1^ s^−1^) account for the excellent electron transport and hole-blocking properties of PO-T2T. For HTLs, poly(3,4-ethylenedioxythiophene):polystyrene sulfonate (PEDOT:PSS) is commonly used, and its work function is ∼5.2 eV^132^. Notably, in terms of hole injection, a large barrier exists between PEDOT:PSS and the perovskite layer with a deeper valence band, especially in green and blue emitters. Fortunately, this predicament can be overcome by employing poly(sodium 4-styrenesulfonate) (PSS-Na) to increase the work function of PEDOT:PSS^45,81,133^. PEDOT:PSS doped with perfluorinated ionomer (PFI) can also achieve similar effects^79,104^. In addition, HTLs with low HOMO levels, such as poly[bis(4-phenyl)(2,4,6-trimethylphenyl)amine] (PTAA), poly(9,9-dioctylfluorene-co-*N*-(4-butylphenyl)-diphenylamine) (TFB), poly(9-vinlycarbazole) (PVK), and poly[bis(4-phenyl)(4-butylphenyl)amine] (poly-TPD), were deposited on PEDOT:PSS to form gradient energy levels for hole injection, which can also achieve charge balance effectively^72,134–136^.

Balanced carrier transport and sufficient radiative recombination center density in perovskite emissive layers are also key to ensuring excellent electrical properties. Here, we summarize the effects of organic cation spacers and grain orientations on the carrier transport and recombination capabilities of quasi-2D perovskite films when the device is operated under a bias voltage. Due to the insulating nature of organic cation spacers, the charge transport of the quasi-2D perovskite films becomes anisotropic and highly restricted, damaging the device performance. Wu et al. replaced large organic molecules with small basic sodium ions (Na^+^) to improve the conductivity of the emissive layers. The Na^+^ could generate amorphous sodium lead bromide (NaPbBr_3_) in the perovskite as spacers to form a nanocrystal-like halide perovskite film (Fig. 8b). High EQE (15.9%) and PLQY (>50%) were achieved by varying the inorganic salt molar ratio and adding trace organic additives to the perovskite^43^. In addition, molecular engineering to control the barrier width of quasi-2D perovskites is another way to optimize the conductivity of the emissive layers. The introduction of a rigid benzimidazole (BIZ) molecule into quasi-2D perovskites resulted in the formation of the novel quasi-2D perovskite (BIZ)_2_(FA)_*n*__−__1_Pb_*n*_Br_3*n*+1_ with reduced barrier width and increased carrier mobility^137^.

Controlling the grain orientations in quasi-2D perovskite films is crucial for efficient carrier transport. Extensive research on the grain orientations of quasi-2D perovskite solar cells will help us systematically study and understand the grain orientations of the emissive layers in quasi-2D PeLEDs. Ideally, vertically oriented films provide a direct path for hole and electron transport. Therefore, by controlling the vertical orientations of the grains in perovskite films, the suppressed out-of-plane charge transport caused by organic cations can be solved^138^. Tsai et al. first reported that vertically oriented films could facilitate efficient charge injection and transport (Fig. 8c). As a result, they achieved efficient quasi-2D PeLEDs with a radiance of 35 W Sr^−1^ cm^−2^ at 744 nm and an ultralow turn-on voltage of 1 V^139^. Different from the above, Tae-Woo Lee et al. reported a strategy to improve the EQEs of (PEA)_2_(CH_3_NH_3_)_*n*__−__1_Pb_*n*_Br_3*n*+1_ PeLEDs by introducing structure-modulated and randomly oriented perovskite emissive layers. The random grain orientations in the quasi-2D perovskite films forced contact between the inorganic layers to improve charge transport and radiative recombination^140^. Consequently, until a clear consensus is reached on the effect of grain orientation on carrier transport, efforts should be intensified.

### Interfacial engineering

In quasi-2D PeLEDs, the physical properties of the bottom interlayers affect the properties of the subsequently deposited perovskite layers, e.g., the crystallinities, morphologies, and defect densities^141^. The perovskite-interlayer interfaces have a complex effect on the performance of quasi-2D PeLEDs and deserve further investigation. Interface defects should be responsible for the low PLQYs of quasi-2D perovskite films deposited on top of the interlayers. Therefore, interlayer modification by interfacial engineering is essential.

The surface wettability of bottom interlayers can affect the crystallization process of the subsequently deposited perovskite films^142^. Zhao et al.^143^ showed that an ultrathin (∼1 nm) layer of lithium fluoride (LiF), an ionic compound with strong polarity, can improve the crystal quality and carrier lifetime of perovskite films on top of the polymeric hole transport layer TFB. The perovskite film formed on the TFB/LiF interface showed larger and more defined crystal grains and reduced pinhole density compared with the perovskite film formed on the oxygen-plasma-treated TFB surface. The LiF layer acted as a useful template for the growth of high-quality perovskite films and enhanced the device performance for green quasi-2D/3D PeLEDs. Similarly, novel NiO_*x*_/LiF HTLs can also avoid luminescence quenching at the surface. The inert LiF intermediate layer (∼1 nm) can effectively passivate the NiO_*x*_ HTL and suppress the exciton quenching induced by the -OH groups on the surface of NiO_*x*_^144^. Functional passivating moieties (such as Lewis base/acid groups), if grafted on interfacial materials, are likely to induce additional healing of surface defects. Zhou et al. used MoO_3_-ammonia deposited on PEDOT:PSS in quasi-2D PeLEDs, which not only facilitated hole injection into the perovskite by reducing the contact barrier but also suppressed exciton quenching at the interface^145,146^.

### Light out-coupling

In Eq. (3), *η*_oc_ represents the fraction of photons extracted from quasi-2D PeLEDs and is generally below 30%. Most photons are trapped inside and lost by generating excess thermal energy^147–149^. Even more detrimental in quasi-2D PeLEDs is the much higher refractive index (~2.6)^16,150^ of perovskite materials than their 3D counterparts (~2.0), which limits *η*_oc_ as predicted by ray-optics theory^151^. Shi et al.^152^ studied the photon loss using systematic optical simulations and showed that quasi-2D PeLEDs could achieve theoretical maximum EQEs of ~20%, which indicated serious photon loss. Generally, the light generated in PeLEDs induces a series of optical modes, including the waveguide mode, surface plasmon polariton (SPP) mode, substrate mode, and out-coupled mode. However, only the out-coupled mode (<20%) is beneficial to the light extraction efficiency (LEE), while the SPP mode (20–30%), waveguide mode (20–30%), substrate mode (10–30%), and parasitic absorption (<10%) are consumed within the device^153^. Therefore, efficiently extracting the waveguide, SPP, and substrate modes is critical to further improve the LEE and EQEs of PeLEDs.

Modulating the morphologies and properties of perovskite films is an important strategy to improve light out-coupling. The perovskite-polymer heterostructure (PPBH) can expand the photon emission escape cone of the emission layer to 32° and reduce the refractive index of the standard halide perovskite. Considering the effects of interference, optical constants, and layer thicknesses, an out-coupling factor of up to ~25% was modeled in quasi-2D PeLEDs^16^. More remarkably, lateral photoluminescence experiments showed that light initially confined as modes waveguided in the PPBH layer can propagate up to 80 µm, along with photoluminescence decay beyond 10 μm. The superlong transverse propagation range indicated a possible contribution from photon recycling (PR) (Fig. 8d). Insight into the relatively small Stokes shifts in perovskites suggested significant levels of reabsorption of emitted photons^12,154^. The PR process can assist light out-coupling by randomizing the direction of photon propagation and redirecting photons from trapped to out-coupled modes^155–157^. Cho et al.^158^ further proved that PR had a significant contribution to the light out-coupling of PeLEDs. In the current device structure, to maximize the benefits of PR, solutions to reduce the electrode area and different filter structures were proposed. Additionally, Cao et al. modulated the morphology of perovskite films. The emission layer spontaneously formed as distinct submicrometer-scale crystal platelets, increasing the light out-coupling factor to 30% ^14^ (Fig. 8e). The submicrometric structure inspired us to conclude that patterning perovskite films may further increase *η*_oc_.

**Table 1:** Performance of quasi-2D PeLEDs (from NIR to blue)

Year	Perovskite materials	Device architecture	EL [nm]	FWHM [nm]	Peak EQE [%]	*L*_max_ [cd m^−^^2^]/ *R*_max_ [Wsr^−^^1^ m^−2^]	*T*_50_ stability	Refs.
*Near-infrared (NIR) quasi-2D PeLEDs*								
2016	(PEA)_2_MA_4_Pb_5_I_16_	ITO/TiO_2_/perov/F8/MoO_3_/Au	750	–	8.8	80^R^	–	^30^
2016	(NMA)_2_FAPb_2_I_6_Br	ITO/ZnO/PEIE/perov/TFB/MoO_*x*_/Au	786	–	11.7	82^R^	2 h @10 mA cm^−2^	^42^
2017	MAPbI_3_·20%FPMAI	ITO/Poly-TPD/perov/TPBi/LiF/Al	749	–	7.9	72^R^	>10 h@3 mA cm^−2^	^71^
2017	MAPbI3·20%BAI	ITO/Poly-TPD/perov/TPBi/LiF/Al	748	–	10.4	30	90 s@5 v	^73^
2018	Cs_0.2_FA_0.8_PbI_2.8_Br_0.2_·20%BAI	ITO/Poly-TPD/perov/TPBi/LiF/Al	752	–	17.6	199^R^	50 min@10 mA cm^−2^	^168^
2018	(NMA)_2_(FA)_*n*__−__1_Pb_*n*_I_3*n*+1_·poly-HEMA	ITO/MZO/PEIE/PPBH/TFB-PFO/MoO_*x*_/Au	~780	~49	20.1	<10^R^	46 h@0.1 mA cm^−2^	^16^
2018	MAPbI_3_·FPMAI	AgNW/Poly-TPD/perov/TPBi/LiF/Al	740	–	13	–	–	^185^
2018	(NMA)_2_FA_*n*__−__1_Pb_*n*_I_3*n*+1_	ITO/PEIE-ZnO/perov/TFB/MoO_*x*_/Al	~790	–	12.7	254^R^	30 min@100 mA cm^−^^2^	^163^
2019	MAPbI_3_·20%PMAI	ITO/Poly-TPD/perov/TPBi/LiF/Al	750	–	15	–	–	^72^
2019	(EDBE)FA_*n*__−__1_Pb_*n*_I_3*n*+1_	ITO/ZnMgO/perov/TFB/MoO_*x*_/Al	804	–	11.4	60^R^	1.3 h@10 mA cm^−2^ 20 h@2 mA cm^−2^	^171^
2019	(BAB)FA_*n*__−__1_Pb_*n*_I_3*n*+1_	ITO/PEIE-ZnO/perov/TFB/MoO_*x*_/Al	776	50	5.2	88.5^R^	>100 h@25 mA cm^−2^	^167^
2020	PbS QDs in (PEA)_2_Cs_2_Pb_3_Br_10_	ITO/PEDOT:PSS/QDLP/TPBi/LiF/Al	980	–	8.1	7.4^R^	>1 h@10 mA cm^−2^	^132^
2020	(NMA)_2_FA_*n*__−__1_Pb_*n*_I_3*n*+1_	Glass/Au/ZnO/PEIE/perov/TFB/MoO_3_/Au	800	35.4	20.2	114.9^R^	–	^29^
*Red quasi-2D PeLEDs*								
2017	(NMA)_2_Cs_*n*__−__1_Pb_*n*_(I/Cl)_3*n*+1_	ITO/ZnO/PEIE/perov/TFB/MoO_*x*_/Au	688	–	3.7	~440	5 h@10 mA cm^−2^	^38^
2017	(PBA)_2_Cs_*n*__−__1_Pb_*n*_I_3*n*+1_	ITO/NiO/Poly-TPD/PVK/perov/TPBi/Ca/Al	683	~34	7.3	~130	–	^72^
2018	(NMA)_2_Cs_*n*__−__1_Pb_*n*_I_3*n*+1_	ITO/ZnO/PEIE/perov/TFB/MoO_*x*_/Au	694		7.3	732	–	^119^
2018	(BA)_2_Cs_*n*__−__1_Pb_*n*_I_3*n*+1_·PEO	ITO/PEDOT:PSS/Poly-TPD/perov/BCP/LiF/Al	638	61	0.41	390	>4 h@3.5 v	^84^
			664	50	2.81	1231		
			680	39	6.23	1392		
			690	36	4.73	186		
2019	(PBA)_2_Cs_*n*__−__1_Pb_*n*_I_3*n*+1_	ITO/PEDOT:PSS/Poly-TPD/PVK/perov/TPBi/LiF/Al	664	(0.72, 0.27)	13.3	968	–	^44^
2020	(PEA)_2_SnI_4_	ITO/PEDOT:PSS/perov/TPBi/LiF/Al.	633	24/(0.706,0.294)	0.3	70		^48^
2020	(PEA)_2_SnI_4_	ITO/PEDOT:PSS/perov/TPBi/LiF/Al	632	21/(0.708,0.292)	5	~100	>15 h@20 cd m^−2^	^112^
*Green quasi-2D PeLEDs*								
2016	(PEA)_2_MA_*n*__−__1_Pb_*n*_Br_3*n*+1_	ITO/Buf-HIL/perov/TPBi/LiF/Al	525	–	4.9(CE)	2935	–	^63^
2017	(PEA)_2_MA_*n*__−__1_Pb_*n*_Br_3*n*+1_	ITO/PEDOT:PSS/perov/TPBi/LiF/Al	526	–	7.4	8400	–	^75^
2017	(PBA)_2_Cs_*n*__−__1_Pb_*n*_Br_3*n*+1_	ITO/NiO/TFB/PVK/perov/TPBi/Ca/Al	514	17	10.4	14,000	–	^72^
2018	(C_8_H_17_NH_3_)_2_FA_*n*__−__1_Pb_*n*_Br_3*n*+1_	ITO/LiF/perov/PO-T2T/Ca/Al	540	–	5	~3000	–	^76^
2018	(OA)_2_FA_*n*__−__1_Pb_*n*_Br_3*n*+1_ FAPbBr_3_ NCs	ITO/PEDOT:PSS/perov/PO-T2T/Ca/Al	528–532	25–26	13	56,143	–	^131^
2018	(BA)_2_FA_2_Pb_3_Br_10_	ITO/NiOx/perov/TPBi/LiF/Al	530–540	–	14.6	24,100	102 min@100 cd m^−2^	^82^
2018	(PEA)_2_FA_2_Pb_3_Br_10_	ITO/m-PEDOT:PSS/perov/TOPO/TPBi/LiF/Al	532	23	14.36	9120	70 min@280 cd m^−2^	^81^
2018	(BA)_2_Cs_*n*__−__1_Pb_*n*_Br_3*n*+1_·PEO	ITO/PEDOT:PSS/perov/TPBi/Ca/Al	514	19–21	8.42	33,533	45 min@100 cd m^−2^	^83^
2018	(PEA)_2_Cs_*n*__−__1_Pb_*n*_Br_3*n*+1_/Cs_4_PbBr_6_	ITO/PEDOT:PSS:PFI/perov/TPBi/LiF/Al	500	24	4.51	3259	72 min@10 mA cm^−2^	^166^
2018	(PEA)_2_Cs_*n*_Pb_*n*_Br_3*n*+1_	ITO/Poly-TPD/PFN/perov/TPBi/LiF/Al	512	22	14.4	23,380	>25 min@3.5 v	^157^
2018	(BIZ)_2_FA_2_Pb_*n*_Br_3*n*+1_	ITO/PVK/perov/TmPyPB/LiF/Al	537	–	7.7	30,000	63 min@1330 cd m^−2^	^137^
2018	(PEA)_2_Cs_*n*__−__1_Pb_*n*_Br_3*n*+1_	ITO/Poly-TPD/perov/TPBi/LiF/Al	514	–	15.5	~20,000	90 min@2 mA cm^−2^	^78^
2018	FAPbBr_3_·DPPABr	ITO/PEDOT:PSS/TFB/perov/TPBi/LiF/Al	526	22	16.3	13,970	–	^135^
2019	(PBA/PA)_2_FA_*n*__−__1_Pb_*n*_Br_3*n*+1_	ITO/PVK:TPD-DDAB/perov/TPBi/CsF/Al	534	25	15.1	8052	–	^85^
2019	(PEA)_2_FA_*n*__−__1_Pb_*n*_Br_3*n*+1_	ITO/PEDOT:PSS-Na/perov/TOPO/TPBi/LiF/Al	532	–	15.4	15,765	–	^133^
2019	(PEA)_2_FA_2_Pb_3_Br_10_	ITO/PEDOT:PSS/perov/TPBi/LiF/Al	525	–	10.6	41,500	9.2 h@10 mA cm^−2^	^156^
2019	CsPbBr_3_·NaBr	ITO/TFB/PVK/perov/TPBi/LiF/Al	518	21.7	15.9	11,560	~2.5 h@150 cd m^−2^	^43^
2019	BA_2_Cs_*n*_Pb_*n*_Br_3*n*+1_	ITO/PEDOT:PSS/PVK/perov/TPBi/Al	506	–	10.1	3810	–	^98^
			513		7.2	11,200		
2019	(PEA)_2_Cs_*n*__−__1_Pb_*n*_Br_3*n*+1_·NaBr	ITO/NiO_*x*_/PVK/perov/TPBi/LiF/Al	512	–	17.4	8353	40 min@100 cd m^−2^	^91^
2020	PEA_2_Cs_2.4_MA_0.6_Pb_4_Br_13_	ITO/PEDOT:PSS:PFI/perov/TPBi/LiF/Al	517	–	14	45,230	3.5 h@4000 cd m^−2^	^79^
2020	(PEA)_2_FA_*n*__−__1_Pb_*n*_Br_3*n*+1_	ITO/PVK/perov/TPBi/LiF/Al	529	23	14.7	37,477	<1 h@0.25 mA cm^−2^	^86^
2020	(PEA)_2_(FA/Cs)_*n*__−__1_Pb_*n*_Br_3*n*+1_	ITO/PEDOT:PSS/perov/TPBi/LiF/Al	528	24	4.5	10,000	42 s@5 V	^136^
2020	(PEA)_2_FA_*n*__−__1_Pb_*n*_Br_3*n*+1_	ITO/PVK/perov/TPBi/LiF/Al	527	21	12.4	~6500	1.5 h@1 mA cm^−2^	^134^
2020	(PEA)_2_Cs_*n*__−__1_Pb_*n*_Br_3*n*+1_	ITO/TFB/LiF/perov/TPBi/LiF/Al	~520	~18	19.1	~50,000	–	^143^
2021	(*p*-FPEA)_2_MA_*n*__−__1_Pb_*n*_Br_3*n*+1_	ITO/PEDOT:PSS/PFNBr/perov/PMMA/TmPyPB/LiF/Al	525	21.2	20.36	82,480	6.5 min@10000 cd m^−2^	^164^
*Blue quasi-2D PeLEDs*								
2016	(PEA)_2_PbBr_4_	ITO/PEDOT: PSS/perov/TPBi/Al/Ca	410	–	0.04	–	–	^194^
2016	(OLA)_2_MA_*n*__−__1_Pb_*n*_Br_3*n*+1_	ITO/PEDOT: PSS/CBP/perov/TPBi/LiF/Al	492	24	0.23	8.5		^88^
			456	18	0.024	1		
			432	40	0.004	1		
2017	(PEOA)_2_MA_*n*__−__1_Pb_*n*_Br_3*n*+1_	ITO/PEDOT: PSS/perov/TPBi/Ba/Al	494	–	1.1	19.5	–	^195^
			462		0.06	1.26		
2017	(4-PBA)_2_PbBr_4_	ITO/PEIE-ZnO/perov/TFB/MoO_*x*_/Al	491	–	0.015	186	–	^90^
2017	(EA)_2_MA_*n*__−__1_Pb_*n*_Br_3*n*+1_	ITO/PEDOT:PSS/perov/TmPyPB/CsF/Al	473	–	2.6	200	–	^89^
2018	(PEA/IPA)_2_(MA/Cs)_*n*__−__1_Pb_*n*_Br_3*n*+1_	ITO/PEDOT:PSS/NiO_*x*_/PVK/perov/TPBi/LiF/Al	490	28	1.5	2480	10 min@10 cd m^−2^ 4 min@20 cd m^−2^ 0.5 min@210 cd m^−2^	^121^
2018	PA_2_Cs_*n*__−__1_Pb_*n*_Br_3*n*+1_	ITO/PEDOT:PSS/perov/TmPyPB/Cs_2_CO_3_/Al	505	26	3.6	7320	30 min@4.8 v	^92^
2019	(BA)_2_Cs_*n*__−__1_Pb_*n*_(Br/Cl)_3*n*+1_	ITO/PEDOT:PSS/PVK/perov/TPBi/Al	465	23	2.4	962	1 min@500 cd m^−2^	^98^
			487	25	6.2	3340	10 min@800 cd m^−2^	
2019	(PBA)_2_Cs_*n*__−__1_Pb_*n*_(Br/Cl)_3*n*+1_	ITO/NiO_*x*_/LiF/perov/TPBi/LiF/Al	490	21	0.52	1446	–	^144^
2019	(PA)_2_Cs_*n*__−__1_Pb_*n*_Br_3*n*+1_	ITO/NiO_*x*_-PSSNa/perov/TPBi/LiF/Al	492	26	1.45	4359	220 min@150 cd m^−2^ 120 min@415 cd m^−2^	^196^
2019	(PEA)_2_Cs_*n*__−__1_Pb_*n*_(Br/Cl)_3*n*+1_	ITO/PEDOT:PSS/perov/TPBi/LiF/Al	485	–	11.0	9040	100 min@100 cd m^−2^	^102^
2019	(PEA)_2_Cs_*n*__−__1_Pb_*n*_(Br/Cl)_3*n*+1_	ITO/PEDOT:PSS/perov/TPBi/LiF/Al	480	21	5.7	3780	10 min@1500 cd m^−2^	^97^
2019	(PEA/P-PDABr_2_)_2_Cs_*n*__−__1_Pb_*n*_Br_3*n*+1_	ITO/PVK/PFI/perov/3TPYMB/Liq/Al	465	25	2.6	211	13.5 min@0.35 mA cm^−2^	^117^
2019	(PEA)_2_(Rb/Cs)_*n*__−__1_Pb_*n*_Br_3*n*+1_	ITO/PEDOT:PSS/perov/TmPyPB/LiF/Al	475	20	1.35	100.6	14.5 min@15 cd m^−2^	^110^
2019	(PEA/NPA)_2_Cs_*n*__−__1_Pb_*n*_Br_3*n*+1_	ITO/PVK/perov/PO-T2T/Liq/Al	485	23	2.62	1200	4.3 min@100 cd m^−2^	^116^
2019	PBABr_2_(Cs_0.7_FA_0.3_PbBr_3_)	ITO/NiO_*x*_/TFB/PVK/perov/TPBi/LiF/Al	483	–	9.5	700	4 min@100 cd m^−2^	^137^
2020	PEA_2_Cs_1.6_MA_0.4_Pb_3_Br_10_·DPPOCl	ITO/PEDOT:PSS:PFI or Poly-TPD/perov/TPBi/LiF/Al	489	18	1.3	5141	51 min@1500 cd m^−2^	^104^
			479	18	5.2	468	90 min@100 cd m^−2^	
2020	(PBA)_2_Cs_*n*__−__1_Pb_*n*_Br_3*n*+1_	ITO/PEDOT:PSS/perov/POT2T/LiF/Al	465	–	2.34	150	232 s@12 cd m^−2^	^115^
			493		5.08	1000		
2020	CsPbBr_3_·GABA	ITO/PEDOT:PSS:PFI/PVK/CsPbBr_3_ QW/TPBi/LiF/Al	478	(0.12, 0.14)	6.3	200	150 s@200 cd m^−2^	^122^
2020	PEA_2_(Cs_1__−__*x*_EA_*x*_PbBr_3_)_2_PbBr_4_	ITO/m-PEDOT:PSS/perov/TPBi/LiF/Al	495	23	13.3	2790	200s@100 cd m^−2^	^45^
			488	25	12.1	2191		
			480	25	4.19	83		
2020	(PEA)_2_Cs_*n*__−__1_Pb_*n*_(Br/Cl)_3*n*+1_·NaBr	ITO/NiO_*x*_/PTAA/PVK/perov/TPBi/LiF/Al	488	18.8	11.7	1511	970 s@100 cd m^−2^	^197^
2020	(PEA/PA)_2_Cs_*n*__−__1_Pb_*n*_Br_3*n*+1_	ITO/PVK/perov/TPBi/LiF/Al	486	25	10.11	~600	81.3 min@0.3 mA cm^−2^	^120^
2020	(Cs/FA/*p*-F-PEA)Pb(Cl/Br)_3_	ITO/PEDOT:PSS/perov/TPBi/LiF/Al	469	(0.125, 0.076)	4.14	451	14.0 min@1 mA cm^−2^	^198^

Modifying the device structure is another strategy to achieve high *η*_oc_. An ultrathin PEDOT:PSS (UT-PEDOT:PSS) HTL was reported to enhance the light extraction efficiency of quasi-2D PeLEDs. An increase in the PEDOT:PSS thickness led to a reduction in the portion of the out-coupled mode because the extinction coefficient of PEDOT:PSS is nonzero in the green light region^159^ (Fig. 8f, g). In another study that made more sense by Wang’s group, the microcavity effect was employed to enhance light extraction. They used a total-reflection Au bottom electrode accompanied by a semitransparent Au top electrode in a simple top-emission (TE) LED device structure. *η*_oc_ thus vastly improved due to the microcavity effect (Fig. 8h), and a high peak EQE of 20.2% in the quasi-2D PeLEDs was achieved^29^. More recently, Chen et al.^153^ designed a rational device structure that utilizes the near-field coupling between different emitters via evanescent fields to extract trapped photons. They can efficiently extract the waveguide mode by photon tunneling and evanescent wave absorption. Simultaneously, the SPP mode can be utilized via reabsorption or reemission processes. This simple and efficient approach led to high-performance white PeLEDs (EQE of >12%) with much-enhanced LEE (over 50%). For more clarity, we summarize the recent reports in the field of PeLEDs on improving the LEE and corresponding strategies (Table 2). Moreover, the corresponding device structures are also included. Research on improving the *η*_oc_ in quasi-2D PeLEDs is just in its infancy. Various valuable strategies have not been effectively used, such as using diffraction gratings, buckling the device and texturing meshed surfaces^160^, prompting researchers to pay more attention to addressing this issue in the future.

**Table 2:** Light extraction efficiency enhancement and corresponding strategies in recent reports on PeLEDs

Year	Emitting materials	Device structure	Light extraction strategy and efficiency	Refs.
2018	(NMA)_2_FA_*n*__−__1_Pb_*n*_I_3*n*+1_ - Polymer	Glass/ITO/MZO/PEIE/PPBH/TFB-PFO/MoOx/Au	Reduced refractive index (1.9) of PPBH; thin emissive layer; LEE: ~25%;	^16^
2018	Cs_0.2_FA_0.8_PbI_2.8_Br_0.2_	Glass/ITO/Poly-TPD/Perovskite/TPBi/Al	Thin emitting layers; EQE 17.6% (IR)	^168^
2018	5-AVA/FAPbI_3_	Glass/ITO/ZnO-PEIE/Organic layer/Perovskite/TFB/MoOx/Au	EQE 20.7% (green); light extraction efficiency from 20% to more than 30%	^17^
2019	BA: CH_3_NH_3_PbBr_3_ (Br-Pero)	Glass/Epoxy/AAM(TiO_2_)/ITO/PEDOT:PSS/Perovskite/F8/Ca/Ag	Nanophotonic substrate (light coupler optical antennas); EQE 17.5% (green); LEE from 10–20% to more than70%;	^199^
2019	Modified CsPbBr_3_	Glass/ITO/moth-eye ZnO/PEDOT:PSS/Perovskite/TPBi/LiF/Al	Moth-eye ZnO injection layer + half-ball lens; EQE 28.2% (green)	^200^
2019	MAPbI_3_	Glass/NHAs/ITO/Poly-TPD/Perovskite/TPBi/LiF/Al	Nanohole array with high-index contrast; peak EQE 14.6% (red/near IR); 1.64 times light extraction enhancement	^201^
2020	(PEA)_2_Cs_*n*__−__1_Pb_*n*_Br_3*n*+1_	ITO/PVK/Perovskite/TPBi/LiF/Al	Photon recycling; photonic structure control; an out-coupling efficiency of 100% is theoretically possible	^159^
2020	MABr modified CsPbBr_3_ PNW	Glass/ITO/PEDOT:PSS/Perovskite PNWs/TPBi/Cathode	Perovskite PNWs; EQE 16% (green); LEE from 10–20% to 40−50%	^202^
2020	3D, quasi-3D, and quasi-2D	Glass/ITO/UT-PEDOT:PSS/Perovskite/B3PYMPM/LiF/Al	Ultrathin PEDOT:PSS; EQE enhancements 42% 87% 111% for 3D, quasi-3D and quasi-2D, respectively;	^160^
2020	(NMA)_2_FA_*n*__−__1_Pb_*n*_I_3*n*+1_	Glass/Au/ZnO/PEIE/MQW-Perovskite/TFB/MoO_3_/Au	Microcavity top-emission; the enhanced microcavity effect; EQE 20.2%	^29^
2021	(PEA/IPA)_2_Cs_*n*__−__1_Pb_*n*_Br_3*n*+1_;	Glass/ITO/NiOx/PVK/Sky-blue Perovskite/TPBi/LiF/Al/Ag/LiF/Red PeNCs	Extract both waveguide and SPP modes	^154^

### Stability of quasi-2D PeLEDs

In addition to improving the efficiency of quasi-2D PeLEDs, it is crucial to address the environmental stability and long-term reliability of materials and devices. Quasi-2D perovskites have better stability than their 3D analogs when facing ambient erosion^161^. The main reason is the addition of large organic cations, which provide a protective barrier, isolating the materials from the outside environment and protecting them from moisture, oxygen, etc.^162^. Although the environmental stability of materials can be improved by judicious selection of large organic cations, the situation is quite different when applied to LEDs. Even in the best case, quasi-2D PeLEDs can only work for a few hundred minutes. However, there is still little consensus in the community about the factors limiting the stability of these devices. In this review, we summarize several possible causes.

Nonradiative Auger recombination is an essential factor for the reduced operational stability of quasi-2D PeLEDs. Quasi-2D PeLEDs suffer from finite maximum brightness and terrible efficiency roll-off, mainly caused by luminescence quenching resulting from nonradiative Auger recombination^163^. We also note that the current density threshold of the Auger recombination in quasi-2D PeLEDs is well below that of their 3D analogs. This can be attributed to the efficient energy transfer in films that exacerbates excessive local carrier concentration and makes them susceptible to Auger recombination. In addition, the large *E*_b_ in quasi-2D perovskites also increases the charge-carrier probability of spatial encounters^164^. Therefore, it is urgent to find effective strategies to suppress Auger recombination. Zou et al.^163^ increased the width of the QWs to suppress Auger recombination. The real cause of the efficiency roll-off and more effective measures to suppress Auger recombination needs to be further pursued by researchers.

**Fig. 9:**
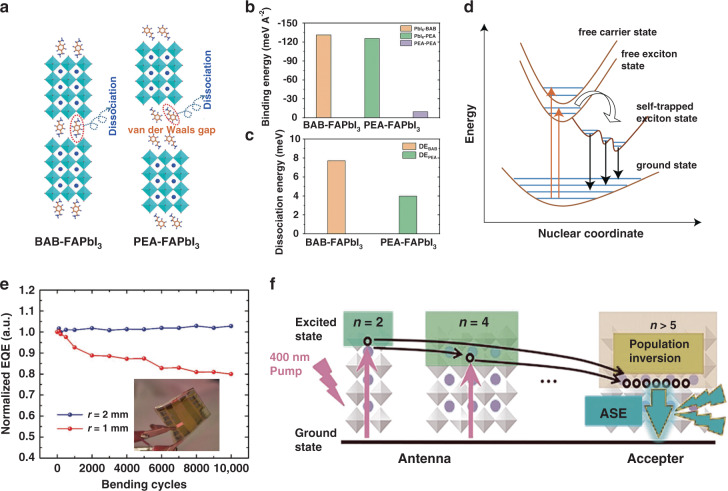
Prospects for quasi-2D perovskite materials and devices. **a** Schematic crystal structures of BAB-FAPbI_3_ and PEA-FAPbI_3_ quasi-2D perovskites. **b** Calculated binding energies of PbI_6_-BAB, PbI_6_-PEA, and PEA-PEA in quasi-2D perovskites. **c** Calculated molecular dissociation energy of the BAB- and PEA-based quasi-2D perovskites. **d** Schematic illustration of the self-trapped exciton emission process. **e** Normalized EQE depending on the 1- and 2-mm bending cycle of flexible quasi-2D PeLEDs doped with FPMAI. Inset: Digital photo of the working flexible quasi-2D PeLEDs doped with FPMAI. **f** Schematic of the amplified spontaneous emission process realized by population inversion in quasi-2D perovskites. Panels **a**–**c** are reprinted from ref. ^167^ with permission from the AAAS. Panel **d** is reprinted from ref. ^203^ with permission from Springer Nature. Panel **e** is reprinted from ref. ^185^ with permission from Wiley. Panel **f** is reprinted from ref. ^188^ with permission from Wiley

Field-induced decomposition of spacer cations under a voltage bias is also a crucial factor affecting the stability and lifetime of quasi-2D PeLEDs. Warby et al. attributed the operational instability to the increased mobility of ammonium ions, which led to the decomposition of spacer cations^165,166^. The field-induced decomposition of the spacer cations and the subsequent spontaneous conversion from quasi-2D into the corresponding 3D phase were considered another reason for the degradation of the operational stability. The dissociation of surface passivating molecules introduced oxygen and water into the lattice structure, leading to further decomposition of the inorganic octahedral scaffold. Therefore, increasing the interaction between large organic cations and inorganic layers is a feasible strategy to improve the operational stability of quasi-2D PeLEDs. Compared with the monoamine cations in RP configurations, the diamine cations in DJ configurations can form a strong interaction with the inorganic layer. Shang et al.^167^ used 1,4-bis(aminomethyl)benzene (BAB), a dicarboxylic acid organic cation with higher dissociation energy, to construct a quasi-2D perovskite with a DJ configuration (Fig. 9a–c). The device showed an operating lifetime of 100 h, almost two orders of magnitude longer than that of the RP configuration. This conclusion was well consistent with the previous study by Yuan et al.^117^.

In addition, some other factors lead to poor device stability. For example, insulating organic long-chain spacer cations result in high internal resistance under a voltage bias, which brings Joule heating and irreversibly destroys the functional layer of the device^168^. Grain orientation can improve the carrier transport in the emissive layers, reducing the internal resistance and Joule heating. Furthermore, field-induced ion migration also affects operational stability. Ion migration can shield the applied electric fields and cause undesirable changes in quasi-2D PeLEDs. In addition, the ions favor locating at interfaces or grain boundaries, leading to changes in interfacial properties and the formation of defect sites. Thus, we note that the degradation mechanisms of quasi-2D PeLEDs seem more complicated than those of other LEDs. However, the currently limited exploration cannot completely address this considerable challenge. Therefore, systematic research, an in-depth understanding of the degradation mechanisms, and continuous exploration of appropriate solutions are necessary.

## Conclusions and perspectives

Owing to their excellent optical and electrical properties, quasi-2D perovskites have flourished in LEDs in merely five years, promising to emulate established technologies such as OLEDs and QLEDs. The state-of-the-art quasi-2D PeLEDs reported have achieved EQEs exceeding 20%. The advances in quasi-2D PeLEDs indicate their bright future in the application scenarios of ultrahigh-definition displays, solid-state lighting, optical communications, etc. However, despite these impressive achievements, quasi-2D perovskite materials still have many unsettled issues that hinder their further development and application. Here, we discuss the prospects for the future development of novel quasi-2D perovskite materials/structures and white-light-emitting quasi-2D devices and the potential applications of quasi-2D perovskite emitters in large-area, printable, and flexible electronics as well as quasi-2D perovskite lasers, aiming to shed light on these promising future prospects.

### Novel quasi-2D perovskite materials and structures

Searching for large organic cations with excellent chemical and physical properties is the key to exploring new quasi-2D perovskite materials. Large organic cations can introduce additional energy levels, thereby affecting the energy transfer process. In addition, the distortion of the inorganic layer caused by organic cations also influences the luminescence properties of the film. Moreover, the synergistic effect of mixed cations can also achieve an ideal phase distribution and promote significant emission of specific spectra, especially pure red and blue emission.

The environmental toxicity of perovskite precursor materials remains an urgent issue. As a regulated substance, the presence of lead in PeLEDs raises concerns due to its toxicity; it has a high solubility in water and readily leaches into the environment^169^. Exploring lead-free perovskite systems is thus of great significance. In the past several years, some lead-free perovskites and perovskite derivatives have been developed in other perovskite systems, such as the tin-based perovskite Cs_2_SnI_6_^111^ and halide double perovskite Cs_2_AgInCl_6_^24^. However, the performance of lead-free perovskite optoelectronic devices still lags far behind that of their lead-based counterparts. Strategies for improving the device performance include inhibiting tin oxidation, optimizing the structure, and synthesizing novel potential lead-free perovskites^170^.

In addition to new materials, the exploration of new quasi-2D perovskite structures is also a significant aspect. Compared with the comprehensive studies on RP phase perovskites, few studies on Dion–Jacobson (DJ) phase perovskites have been conducted. The diamine cations in DJ configurations can form a strong interaction with the inorganic layer and effectively reduce the distortion of the octahedron, which shows potential for efficient and stable optoelectronic devices^117,167,171^. Other newly developed perovskite structures, e.g., the Aurivillius (AV) phase^172^ and the alternating cations in the interlayer space (ACI) phase^74^, display unique photoelectric properties and deserve to be applied in quasi-2D PeLEDs.

### White-light-emitting quasi-2D PeLEDs

White color EL is highly desirable for practical applications in lighting and photo-communication. EL devices of white-light emission are generally achieved in three architectures, single emissive layer LEDs, multiple emissive layer LEDs, and hybrid LEDs combined with color down-conversion emitters and blue/ultraviolet (UV) chips^173^. The common single emissive layer white LEDs can be produced by using a single white luminescent material or a set of distinct colored luminaries. A single material with efficient and stable white-light emission is an ideal choice for lighting applications, but it is difficult for a single material to achieve photon emission covering the entire visible spectrum. A promising strategy is to use self-trapped excitons (STEs) for white-light emission^24^. In STE emission, the free excitons quickly relax to the self-trapped states of different energies in the bandgap, yielding white-light emission, which was widely observed in 2D perovskites (Fig. 9d).

In addition, quasi-2D perovskites offer great opportunities for direct white-light emission^174^. However, the energy transfer in quasi-2D perovskites becomes a limiting factor for white-light emission because the emission tends to be dominated by the smallest bandgap domains, yet a broadband spectrum is required for white-light emission. Incorporating a dual emitting layer or a tandem configuration into quasi-2D PeLEDs may be an effective approach to increase the spatial and physical separation and reduce energy transfer^175^. Many blind areas still exist in white quasi-2D PeLEDs, and more efforts are required to improve the performance of the devices.

### Technologies for commercial applications

The improved device efficiency and stability inspired researchers to push quasi-2D PeLEDs into commercialization. Large-area, printable, and flexible manufacturing technologies are thus regarded as the next challenges for large-scale commercialization of quasi-2D PeLEDs^176^.

Large-area quasi-2D PeLEDs are necessary when considering their applications in next-generation displays, solid-state lighting, and medical imaging. However, severe performance damage arises when increasing the active area. Controlling the formation of a large-area film is a prerequisite for high-performance large-area PeLEDs^177,178^. Recently, Wang et al. attributed the performance degradation of a large-area device to the defects found in perovskite films. These defects emerged from thermal convection during solvent evaporation and electronic traps formed during perovskite crystallization^179^. They thus raised a molecular modification strategy that eliminates pinholes in perovskite layers by controlling the dynamics of film formation. Simultaneously, Br species can passivate defects in perovskite films, thereby preventing nonradiative recombination. The quasi-2D perovskite films with high PLQY and nucleation density show unique potential for fabricating large-area LED devices. However, few reports on large-area quasi-2D PeLEDs can be found. Kim et al. developed efficient and large-area benzylammonium (BA)_2_Cs_*n*__−__1_Pb_*n*_Br_3*n*+1_ quasi-2D PeLEDs by using the hot-casting method. They proposed that the thermal energy of the substrate reduced the surface tension between the perovskite precursor solution and the substrate. This hot-casting strategy indeed delivered a perovskite film with high crystallinity and fewer pinholes and cracks. However, the performance of large-area (12.8 cm^2^) quasi-2D PeLEDs is still far below that of small-area devices reported^180^. We are convinced that large-area quasi-2D PeLEDs will flourish more in the future by precisely controlling the crystallization kinetics.

With the rise of wearable electronics, curved and foldable displays, etc., the fabrication of flexible devices has also been important research topic^181–183^. Thus, flexible PeLEDs are gradually gaining more attention, mainly covering flexible substrate materials, flexible emitting layer technology, and flexible film encapsulation technology. High-performance flexible PeLEDs have also been fabricated on various lightweight substrates, such as carbon nanotubes and silver nanowires^184,185^. These devices exhibit excellent mechanical robustness with negligible performance loss after up to 10 000 cycles of bending tests^185^. In quasi-2D perovskites, bulky organo-ammonium halide additives may help both passivate surface traps and improve flexibility. Zhao et al. reported flexible quasi-2D PeLEDs by introducing a proper additive to improve both the optoelectronic and mechanical properties of the active film. This strategy yielded highly efficient, robust, and flexible quasi-2D PeLEDs with EQE up to 13% and no degradation after bending for 10 000 cycles at a radius of 2 mm^185^ (Fig. 9e).

Printing technology is an integral part of enabling the scale-up production of quasi-2D PeLEDs. In addition, quasi-2D perovskite films are compatible with low-temperature solution-based manufacturing techniques, such as inkjet, roll-to-roll, and 3D printing, also providing great potential in large-area and flexible electronics. To date, among the patterning methods of perovskite materials, inkjet printing is particularly attractive due to its non-contact process, direct writing and plate making, mask-free nature, and flexible substrate^186,187^. Recently, Jia et al. used inkjet printing technology to prepare a quasi-2D perovskite embedded in polymers and successfully constructed luminous patterns/pictures on the polymer substrate. The composite combined the inherent stability of the quasi-2D perovskite and the outstanding barrier property of polyvinyl chloride (PVC), obtaining excellent resistance to abrasion, air, water, light irradiation, etc., and had broad prospects for application in large-area fluorescent billboards^187^. The available strategies for the scale-up production of large-area/flexible/printable quasi-2D PeLEDs are still limited, which poses significant challenges for commercial applications. Therefore, extensive research should be conducted on manufacturing technologies prior to large-scale commercial applications.

### Quasi-2D perovskite lasers

Amplified spontaneous emission (ASE) and optically pumped pulsed lasing with low ASE and lasing thresholds have been realized with a wide range of perovskite gain media. Quasi-2D perovskites with unique properties, e.g., good stability, high *E*_b_, and natural QW architectures, are better gain media than 3D perovskites for laser applications^77,188–191^. The effective ASE of a quasi-2D perovskite is relative to its carriers accumulated by energy transfer, which achieves higher population inversion (Fig. 9f). An optically pumped (NMA)_2_(FA)Pb_2_Br_7_ quasi-2D perovskite laser with a low ASE threshold carrier density (*ρ*_ASE_ = 6.3 × 10^17^ cm^−3^) under threshold fluence (8.5 ± 0.5 μJ cm^−2^) was demonstrated, and this density was two times smaller than that of the 3D CH_3_NH_3_PbI_3_ perovskite^192^. Since then, optically pumped quasi-2D perovskite lasers have been gradually reported. However, electrically pumped lasers have not yet been reported. The behavior of quasi-2D perovskites under the intense electrical excitation required for electrically pumped lasing remains unexplored. Recently, Qin et al. indicated that singlet–triplet exciton annihilation (STA) is a possible intrinsic mechanism causing lasing death. By using a distributed-feedback cavity with a high-quality factor and applying triplet management strategies, they achieved stable green quasi-2D perovskite lasers under continuous-wave lasing (CW lasing) in the air at room temperature. CW lasing is popular for practical applications in high-density integrated optoelectronic devices and is a crucial step towards electrically pumped lasers, which would pave the way to realizing future current-injection perovskite lasers^193^.

To conclude, we have summarized the fundamental requirements for approaching high-performance quasi-2D PeLEDs from two aspects, the materials and devices. Simultaneously, we have highlighted some key challenges ahead in quasi-2D PeLEDs, e.g., high-performance pure red and blue PeLEDs, long-term operational stability, and environmental safety. In our opinion, an interdisciplinary approach may be proposed to overcome these challenges and create large-scale commercial routes. Finally, we have discussed promising research directions and innovations in developing high-performance and stable quasi-2D materials and devices in the near future. We believe that quasi-2D PeLEDs will have unique advantages in future commercial applications. We hope that our review article will provide broad and comprehensive perspectives for researchers to deepen the development of quasi-2D perovskite materials and devices.
